# Size-resolved fungal bioaerosol diversity over an Indian agricultural field and their ecosystem-health implications

**DOI:** 10.3389/fmicb.2025.1648820

**Published:** 2025-12-03

**Authors:** Emil Varghese, Sarayu Krishnamoorthy, T. K. Hredhya, Kiran Kumari, B. K. Bhattacharya, S. S. Kundu, Jonali Goswami, Shweta Yadav, Rama Shanker Verma, R. Ravikrishna, Sachin S. Gunthe

**Affiliations:** 1 EE Division, Department of Civil Engineering, Indian Institute of Technology Madras, Chennai, India; 2 Center for Atmospheric and Climate Sciences, Indian Institute of Technology Madras, Chennai, India; 3 Department of Environmental Sciences, Central University of Jammu, Samba, India; 4 Department of Environmental Sciences, Government Degree College, Billawar, India; 5 Space Applications Centre, Indian Space Research Organization, Ahmedabad, India; 6 North Eastern Space Applications Centre, Umiam, Meghalaya, India; 7 Department of Biotechnology, Indian Institute of Technology Madras, Chennai, India; 8 Department of Chemical Engineering, Indian Institute of Technology Madras, Chennai, India

**Keywords:** fungal pathogens, environmental strains, useful fungi, medicinal fungi, next-generation sequencing

## Abstract

Particle size is one of the important characteristics of bioaerosols that influences their fate and transport. This study investigates the characterization and size-resolved fungal bioaerosol diversity at an agricultural field in northern India during the winter season. The size-resolved bioaerosol samples were collected using a Micro-Orifice Uniform Deposition Impactor (MOUDI) in two phases: the onset of winter (phase 1) and the end of winter (phase 2), and Next-Generation Sequencing (NGS) was used to identify bioaerosol diversity up to the species level. *Ascomycota* was the predominant phylum in both phases, with *Aspergillus penicillioides* being the dominant species in phase 1 and *Mycosphaerella tassiana* in phase 2. The size range 1–1.8 μm exhibited higher diversity (*H* =3.7 and 2.0 in phases 1 and 2, respectively) and evenness (*E_h_* = 0.9 and 0.5 in phases 1 and 2, respectively), while the 5.6–10 μm range has the highest dominance (*D* = 0.4 and 0.5 in phases 1 and 2, respectively). A total of 189 Operational Taxonomic Units (OTUs) were identified in phase 1 and 128 OTUs in phase 2, classified into ‘pathogenic’ and ‘beneficial/useful’ categories to study their role in ecosystem-health interactions. Species such as *Aspergillus flavus*, *Aspergillus niger*, *Macrophomina phaseolina*, and *Penicillium citrinum* were identified as affecting multiple crop hosts, highlighting the potential for multiple crop yield loss. The size range 1–8-3.2 μm had the highest number of species in phase 1, while 3.2–5.6 and 5.6–10 μm were predominant in phase 2 for plant pathogens. The 1.8–3.2 μm range also had the highest number of potential human pathogens in both phases. This study is the first to explain fungal bioaerosol diversity in an agricultural field based on size and its role in ecosystem-health interaction. These findings emphasize the importance of monitoring and managing fungal bioaerosols to protect agricultural productivity and human health.

## 1 Introduction

Fungi, well-known pathogenic microbes, comprise several yeast species, mushrooms, molds, and more (Hawksworth and Lücking, 2017; Taylor et al., 2014; Woo et al., 2018). It is estimated that the annual emission rate of fungal bioaerosols (such as spores and their various structural segments) from various surfaces and substrates ranges from 28 to 50 Tg a^−1^ (Buée et al., 2009; Elbert et al., 2007; Fröhlich-Nowoisky et al., 2009; Heald and Spracklen, 2009; Tedersoo et al., 2014). During its course in the atmosphere, fungal bioaerosols pose a significant negative impact on agriculture, ecosystem, and human health on local, regional, and global scales, by the dispersion of genetic material, causing a serious threat to humans, animals, and plants, leading to lethal infectious diseases and allergies (Varghese et al., 2024; Krishnamoorthy et al., 2020; Priyamvada et al., 2017a, 2017b; Valsan et al., 2016; Yadav et al., 2020; Fisher et al., 2012; Fröhlich-Nowoisky et al., 2016 and references therein). The ability of fungi to survive independently and the rising number of diseases caused by them have attracted the researcher’s attention to study their pathogenic effects on crops, which hampers a country’s food security (Fisher et al., 2012; Varghese et al., 2024). Also, several fungal plant diseases have been reported worldwide that could cause even 100% crop losses (Després et al., 2012). Concurrently, various fungal propagules and their toxins present in the bioaerosols have been repeatedly reported to cause a wide variety of human infections (Brown et al., 2012; Fröhlich-Nowoisky et al., 2016; Goudarzi et al., 2017; Jaenicke, 2005; Krishnamoorthy et al., 2020; Laumbach and Kipen, 2005; Priyamvada et al., 2017b).

Researchers worldwide have highlighted the emergence of human pathogenic fungal species, attributing this phenomenon to environmental stressors experienced by the fungi. These stresses, alongside the fungi’s ability to develop resistance to commonly used fungicides, have resulted in fungal species that are increasingly pathogenic. These fungi can overcome both host defence mechanisms and the drugs used for treatment (Fisher et al., 2012; Rokas, 2022). This growing threat has been emphasized by Pfaller (2012), who warned of the significant risks posed by these emerging fungal pathogens, especially to high-risk patients such as those undergoing treatment, immunocompromised individuals, and patients on immunosuppressive therapies. The most common fungal allergens belong to *Aspergillus*, *Cladosporium*, *Alternaria*, and *Penicillium* (Priyamvada et al., 2017a,b; Fennelly et al., 2017). Hence, to detect and monitor the infectious diseases caused by microbes, two data sources, ‘ProMED’ (the Program for Monitoring Emerging Diseases) and ‘HealthMap’, have been developed globally (Fisher et al., 2012). It has been reported that the most significant number of animal-infecting and plant-infecting fungal alerts have been reported in the United States and the Indian subcontinent, respectively. Over India, during the last two decades, approximately 48% of the alerts reported by ProMED were of fungal related diseases which affected nearly 39 varieties of crops, and *Puccinia striiformis* and *Pyricularia oryzae* affecting wheat and rice hosts, respectively, are the species that were reported multiple times (Yadav et al., 2020).

Many attempts have been made worldwide to address the aerosolization properties, dispersion, deposition, and the adverse implications caused by the fungal propagules on the ecosystem health and climate (Calhim et al., 2018; Elbert et al., 2007; Fröhlich-Nowoisky et al., 2009; Krishnamoorthy et al., 2020; Priyamvada et al., 2017a, 2017b; Valsan et al., 2016; Woo et al., 2018). The particle size, one of the most important characteristics of the fungal bioaerosols, plays a vital role in fungal fate and transport, deposition in the respiratory system, settling and deposition on the Earth’s surface, resuspension to air, penetration into buildings, and pathogenicity potential to cause diseases in plants (Gat et al., 2021; Tanaka et al., 2020; Thomas, 2013; Wang and Lin, 2012; Yamamoto et al., 2012, 2014). Fungi have unique morphological characteristics, which result in effective aerosolisation and long-distance transport of the spores (Yadav et al., 2020). Fröhlich-Nowoisky et al. (2009) have reported that more human pathogens and allergens are found in the fine fraction (< 3 μm) and plant pathogens in the coarse fraction. Therefore, the size and shape-dependent understanding and behavior of the fungal bioaerosols will not only help to delineate their impacts from other types of bioaerosols but also improve our understanding of the specificity of the role of fungal bioaerosols in ecosystem health (Wang and Lin, 2012).

As the threat caused by fungi to plant and animal biodiversity has been increasing in recent years, several studies are now being carried out worldwide to understand the pathogenic virulence of fungal bioaerosols on crops, plants, animals, insects, and human health (Calhim et al., 2018; Elbert et al., 2007; Fisher et al., 2012; Fröhlich-Nowoisky et al., 2009; Krishnamoorthy et al., 2020; Priyamvada et al., 2017a, 2017b; Valsan et al., 2016; Woo et al., 2018). Advanced studies involving next-generation sequencing have replaced the culture-dependent studies to explore the finer details of the pathogenicity of the bioaerosols (Baldrian et al., 2022; Nilsson et al., 2019; Palmero et al., 2011; Shelton et al., 2002; Tedersoo et al., 2014; Woo et al., 2018). Various tools like FUNGuild have also been introduced to taxonomically parse fungal OTUs by ecological guild independent of sequencing platform or analysis pipeline (Nguyen et al., 2016).

In this study, we investigate the size-resolved diversity of the fungal bioaerosols present in an agricultural field in north India, during the winter season. Further, their ‘pathogenic’ and ‘beneficial/useful’ roles on the plants, humans, and the environment have been studied in detail by analyzing and reviewing the available literature, emphasizing their impact on ecosystem health based on size-resolved diversity. Our analysis generates new insights about the size-resolved diversity of bioaerosols over an agricultural field and their ecosystem-health implications.

## 2 Materials and methods

### 2.1 Study site and bioaerosol sampling

The study site is a wheat crop field (Gurdaspur, Punjab; 32°2′21” N and 75°23′11″E) in India, which is surrounded by horticultural fields growing vegetables and fruits. The size-resolved air samples were collected on glass microfiber filter papers (47 mm diameter, Whatman grade GF/C) using a 10-stage Micro-Orifice Uniform Deposition Impactor MOUDI II 120R, TSI Inc., USA. MOUDI has an inlet with a nominal cut-off of 18 μm and ten different stages for collecting uniformly deposited particles with a nominal cut-off of 10, 5.6, 3.2, 1.8, 1.0, 0.56, 0.32, 0.18, 0.10, and 0.056 μm when operated at a flow rate of 30 lpm. The inlet of the impactor was positioned at a height of 2 meters above ground level, corresponding to the typical human breathing level. The sampling was carried out for 70 h each during the winter season in India in two phases - phase 1 to cover the onset of the winter season (initial two-leaf stage of wheat crop; December 2019) and phase 2 to cover the end of the winter season (harvest period of wheat crop; March 2020). During the winter season, daytime temperatures average around 10 °C, while nighttime temperatures drop to a chilly 2 °C. The prevailing wind direction was predominantly northwesterly, bringing in continental air masses that were dry and characterized by low relative humidity - conditions favorable for the long-distance transport and atmospheric persistence of dust particles and bioaerosols. Another set of samples was collected using a 2-stage sampler as described by Valsan et al. (2015) on the nucleopore membrane filters of 25 mm diameter with a pore size of 0.2 and 5 μm to observe the morphological details of the bioaerosols.

### 2.2 DNA isolation and sequencing

The fungal DNA was isolated from each filter paper using the ZR Quick-DNA™ Fungal/Bacterial Miniprep Kit (Zymo Research, USA). The Internal Transcribed Region (ITR) of the extracted DNA was amplified using the primers forward - GCATCGATGAAGAACGCAGC and reverse – TCCTCCGCTTATTGATATGC using the PCR (Polymerase chain reaction) plate of the thermocycler (SureCycler 8,800, Agilent Technologies Inc., USA). The PCR amplicons were then sequenced using the Next-Generation Sequencing (NGS) method in the Illumina MiSeq platform 2 × 300 bp (forward and reverse sequencing) chemistry to generate ≈1 lakh reads per sample. The DNA sequences obtained from the Illumina MiSeq platform were analyzed using the open-source bioinformatics pipeline QIIME™ 2 (Quantitative Insights into Microbial Ecology) (Caporaso et al., 2010) and the Operational Taxonomic Units (OTUs) were picked using the UCLUST algorithm from the UNITE database (version 7.2). The detailed DNA isolation, NGS, and analysis protocol is mentioned in Varghese et al. (2024).

### 2.3 Biodiversity analysis

The size-resolved fungal diversity of the OTUs identified at the species level is visualized in terms of alpha and beta diversity. The alpha diversity (diversity within the ecosystem) is observed in terms of various parameters such as species richness, relative abundance, Shannon-Wiener diversity index (*H*) (Yadav et al., 2020), Shannon’s evenness of equitability (E_h_), and Simpson’s dominance index (*D*). The calculated alpha diversity indices are plotted using Python libraries such as “pandas,” “seaborn,” “numpy,” and “matplotlib” in the open-source web application Jupyter Notebook (V.6.0.3). The beta diversity (the diversity between the various stages) is expressed using the Principal Coordinate Analysis (*PCoA*). For the obtained OTUs, the eigenvalues for the *PCoA* are estimated with Bray-Curtis distance, and the values are aligned in a two-dimensional space using the packages “vegan” and “ape” of the R programming language [version 4.1.0 (2021-05-18)].

### 2.4 Morphological characterization using SEM analysis

The filter paper samples collected for morphological characterization were cut into 1 cm^2^ pieces, sputter-coated with gold, and imaged using a Hitachi S-4800 Scanning Electron Microscope (SEM) equipped with EDX/EDS at the Chemical Engineering Department, Indian Institute of Technology Madras, Chennai, India. Based on the EDX results, particles with a relative contribution of Carbon (C), Oxygen (O), and Nitrogen (N) greater than 90% were classified as bioaerosols (Valsan et al., 2015), and the images were captured.

## 3 Results

### 3.1 Diversity of fungal bioaerosols

During the DNA sequencing, fungal bioaerosols were identified in five size ranges: 1–1.8 μm (Stage 5 of MOUDI), 1.8–3.2 μm (Stage 4 of MOUDI), 3.2–5.6 μm (Stage 3 of MOUDI), 5.6–10 μm (Stage 2 of MOUDI), and 10–18 μm (Stage 1 of MOUDI). The cumulative number of DNA sequences observed from the samples was 684,827 in phase 1 (onset of winter) and 501,672 in phase 2 (end of winter). In phase 1, 24.2% of the total DNA sequences were identified up to the species level, identifying 189 species-level OTUs. In phase 2, 21.8% of the DNA sequences were identified up to the species level, resulting in 128 OTUs. In phase 1, *Aspergillus penicillioides* was the most dominant species, constituting 37.1% of the species-level OTUs, followed by *Aspergillus flavus* at 8.7%. Other significant species observed in phase 1 included *Bipolaris melinidis* (5.9%), *Schizophyllum commune* (5.6%), *Curvularia lunata* (5.4%), *Rhizopus arrhizus* (3.9%), *Ustilaginoidea virens* (3.6%), *Periconia digitata* (3.0%), *Coprinopsis laanii* (2.4%), and *Curvularia hawaiiensis* (2.3%). In phase 2, *Mycosphaerella tassiana* predominated, making up 66.4% of the sequences, followed by *Aspergillus penicillioides* (9.6%), *Tilletiopsis washingtonensis* (7.9%), *Aspergillus flavus* (2.3%), *Ruinenia clavata* (2.1%), *Blumeria graminis* (2.0%), and *Curvularia lunata* (1.1%).

**Figure 1:**
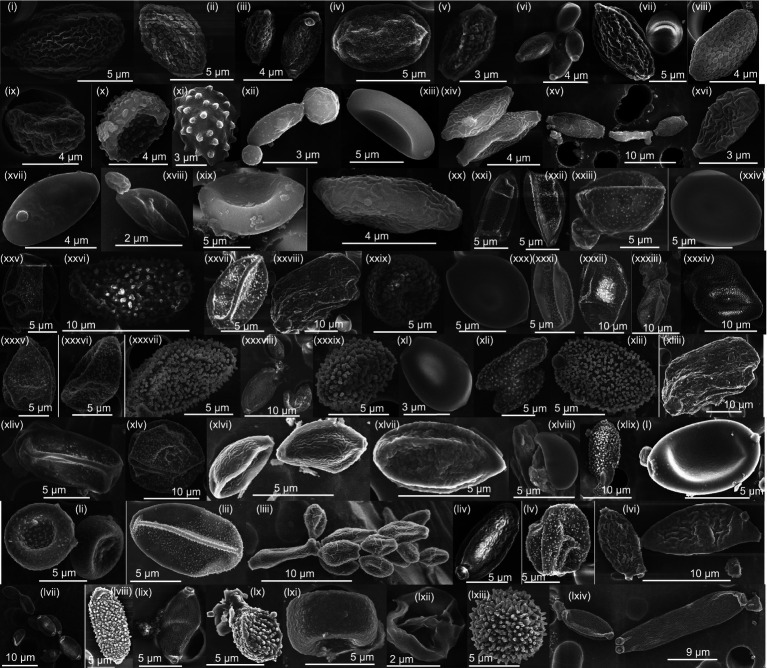
SEM images confirming the presence of fungal bioaerosols covering a wide size range, explaining the broad fungal size distribution.

The species-level identified OTUs belonged to five major phyla: *Ascomycota*, *Basidiomycota*, *Mucoromycota*, *Mortierellomycota*, and *Chytridiomycota*. In both phases, *Ascomycota* was predominant, with 81.4% in phase 1 and 86.0% in phase 2. This was followed by *Basidiomycota* (14.5% in phase 1 and 13.8% in phase 2), *Mucoromycota* (4.02% in Phase 1 and 0.13% in phase 2), *Mortierellomycota* (0.012% in phase 1 and 0.008% in phase 2), and *Chytridiomycota* (0.001% in both phase 1 and phase 2). The family-level taxonomic classification of the species-level identified OTUs comprised 104 families in phase 1 and 70 families in phase 2. In phase 1, *Aspergillaceae* dominated, constituting 52.3% of the species-level OTUs, and in phase 2, *Mycosphaerellaceae* was predominant, making up 66.4%. Figure 1 shows the SEM images of the fungal bioaerosols collected from the agricultural field during phases 1 and 2. Based on morphology, spores from *Ascomycota* and *Basidiomycota* were identified.

In phase 1, out of the 104 family OTUs, 21 OTUs were observed in all five size ranges, and in Phase 2, 23 out of 70 family-level OTUs were present in all five size ranges. The highest number of family-level OTUs was observed in the size range of 1.8–3.2 μm in both phases (77 OTUs in phase 1 and 45 OTUs in phase 2), followed by 3.2–5.6 μm in phase 1 (61 OTUs). In phase 2, the size ranges 3.2–5.6 μm and 5.6–10 μm had 44 OTUs each. Table 1 shows the size-resolved diversity of species-level OTUs, expressed as diversity indices. The phase 1 has a higher diversity (*H* = 2.7) than phase 2 (*H* = 1.5). This was due to the higher richness and abundance in phase 1 (189 OTUs and 165,493 DNA sequences) compared to phase 2 (128 OTUs and 109,161 DNA sequences). The evenness is higher in phase 1 (*E_h_* = 0.5), and dominance is higher in phase 2 (*D* = 0.5). The size range 1–1.8 μm has higher diversity (*H* = 3.7 and 2.0 in phases 1 and 2, respectively) and evenness (*E_h_* = 0.9 and 0.5 in phases 1 and 2, respectively) in both the phases, while the size range 5.6–10 μm has the highest dominance (*D* = 0.4 and 0.5 in phases 1 and 2, respectively).

**Table 1:** Size-resolved diversity of fungal bioaerosols, identified at the species level, during two phases.

Size range	Shannon diversity (H)		Evenness (E_h_)		Simpson dominance (D)	
	Phase 1	Phase 2	Phase 1	Phase 2	Phase 1	Phase 2
1.0–1.8 μm	3.7	2.0	0.9	0.5	0.0	0.2
1.8–3.2 μm	2.8	1.9	0.6	0.4	0.1	0.4
3.2–5.6 μm	3.1	1.3	0.7	0.3	0.1	0.5
5.6–10.0 μm	1.6	1.3	0.4	0.3	0.4	0.5
10.0–18.0 μm	2.7	1.6	0.6	0.4	0.1	0.4

### 3.2 Fungal bioaerosol characterization and their size-resolved diversity

The species-level identified fungal OTUs are classified into two categories: ‘pathogenic’ and ‘beneficial/useful’ based on their ecosystem-health implications. Supplementary Tables S1, S2 list various species identified as ‘pathogenic’ and ‘beneficial/useful’, respectively, based on the literature review. ‘Pathogenic’ refers to the fungal species that cause disease in various hosts such as crops, plants, insects, nematodes and humans, while the ‘beneficial/useful’ refers to the fungal species beneficial in biotechnology, industry, medicine, as well as saprophytic/environmental strains or edible mushrooms. The following sections discuss the size-resolved diversity of these categories.

#### 3.2.1 Size-resolved diversity of crop pathogens

**Figure 2:**
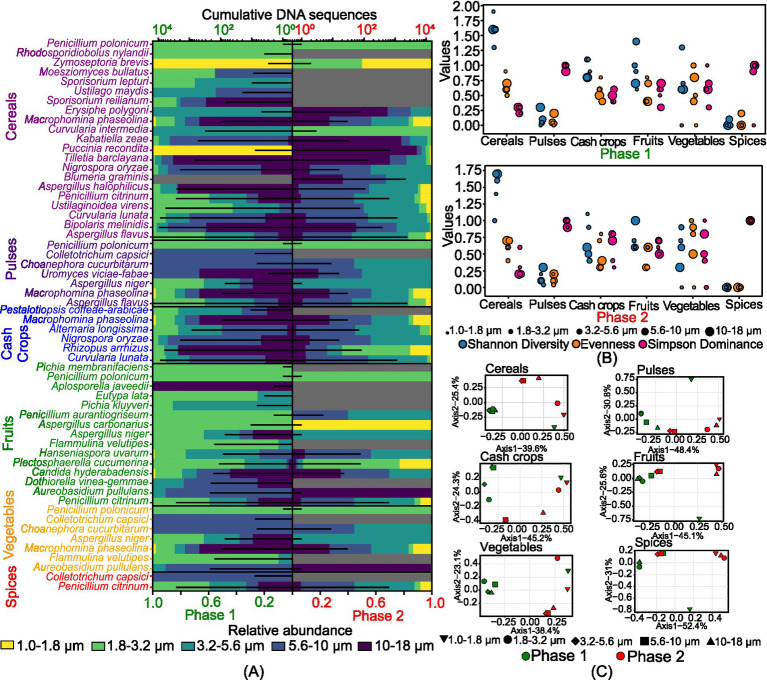
Size-resolved diversity of species identified as crop pathogens during phase 1 and phase 2: **(A)** cumulative DNA sequences and the size-resolved relative abundance; **(B)** size-resolved diversity explained through the various diversity indices like Shannon diversity index, evenness, and Simpson’s dominance; and **(C)** inter-size range diversity.

Figure 2 illustrates the size-resolved diversity of various crop pathogens, classified into cereals, pulses, cash crops, fruits, vegetables, and spices based on their potential hosts. We observed 21 potential cereal pathogens, 7 pulse pathogens, 6 cash crop pathogens, 15 fruit pathogens, 7 vegetable pathogens, and 2 spice pathogens (Figure 2A). The number of DNA sequences for cereals was the least in the 1–1.8 μm size range in both phases. Pulses showed a high number of species in the 5.6–10 μm size range in phase 1 and 3.2–5.6 μm in phase 2. Cash crop species were mainly observed in size ranges greater than 1.8 μm in phase 1 and greater than 3.2 μm in phase 2. For fruits, 15 species were identified, with 12 species in the 1.8–3.2 μm size range and 10 species in the 3.2–5.6 μm size range. Vegetables had the maximum number of species in the 5.6–10 μm size range in phase 1, while the 3.2–5.6 μm size range dominated in phase 2. Both species of spices pathogens were present in the 5.6–10 μm size range in phase 1. Figure 2B shows that cereals exhibited the highest diversity, high evenness, and low dominance among crop pathogens. In phase 1, cereal pathogens showed high diversity in the 1–1.8 μm size range (*H*=1.9), while in phase 2, the 5.6–10 μm and 10–18 μm size ranges were highly diverse (*H*=1.7). Pulse pathogens had low diversity in both phases, with the maximum diversity observed in the 10–18 μm size range (*H*=0.3). Cash crops pathogens displayed moderate diversity and evenness across all size ranges. Fruits pathogens showed unique intra-community structures in each size range during phase 1, but similar diversity indices across multiple size ranges in phase 2. Vegetable pathogens had highly variable diversity, with the highest diversity in the 3.2–5.6 μm size range (*H*=1.3) in phase 1 and (*H*=0.9) in phase 2. Spice pathogens exhibited relatively low diversity in both phases due to the presence of only a few species identified in this category.

Figure 2C presents the Principal Coordinate Analysis (*PCoA*), revealing distinct inter-community structures among pathogenic fungi across different crop categories. For cereal pathogens, phase 1 showed less correlation in the 1–1.8 μm size range, while phase 2 had overlapping communities in the 3.2–5.6 μm and 5.6–10 μm ranges. Pulse pathogens exhibited overlapping species in the 1.8–3.2 μm and 3.2–5.6 μm ranges in phase 1, with unique compositions in the 1.0–1.8 μm range. In phase 2, unique compositions were specific to each size range, with similar communities in the 1.0–1.8 μm, 1.8–3.2 μm, and 10–18 μm ranges. Cash crop pathogens had size-specific compositions, except for overlapping communities in the 5.6–10 μm and 10–18 μm ranges in phase 1, and 3.2–5.6 μm and 5.6–10 μm ranges in phase 2. Fruit pathogens showed similar structures across all size ranges in phase 1, except for 10–18 μm, and overlapping species in the 3.2–5.6 μm and 5.6–10 μm ranges in phase 2. Vegetable pathogens had similar populations across all size ranges in phase 1 but diverse populations with overlapping species in the 3.2–5.6 μm and 5.6–10 μm ranges in phase 2. Spice pathogens exhibited distinct compositions in the 5.6–10 μm range in phase 1 and similar structures in the 3.2–5.6 μm and 5.6–10 μm ranges in phase 2.

#### 3.2.2 Size-resolved diversity of plant pathogens

**Figure 3:**
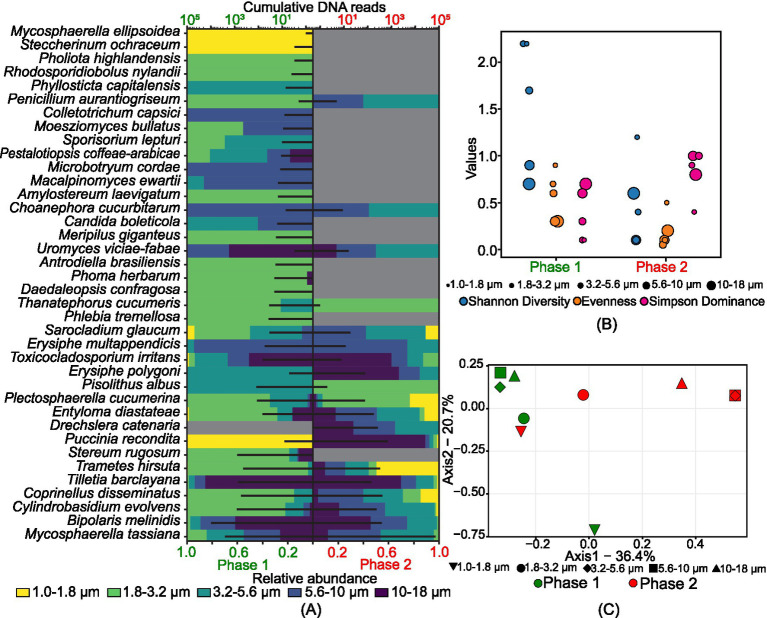
Size-resolved diversity of species identified as plant pathogens during phase 1 and phase 2: **(A)** cumulative DNA sequences and the size-resolved relative abundance; **(B)** size-resolved diversity explained through the various diversity indices like Shannon diversity index, evenness, and Simpson’s dominance; and **(C)** inter-size range diversity.

Figure 3 identifies 38 plant pathogens (excluding crops), with *Bipolaris melinidis* being the most predominant in phase 1 and *Mycosphaerella tassiana* dominant in both phases. In phase 1, 24 species were present in the 1.8–3.2 μm size range, while in phase 2, 17 species each were observed in the 3.2–5.6 μm and 5.6–10 μm size ranges, indicating a shift from smaller to larger size ranges. The Shannon diversity index (*H*) shows highly variable diversity in phase 1 compared to phase 2, with moderate evenness (Figure. 3B). Figure 3C illustrates the diverse nature of plant pathogens across different size ranges in both phases, except for overlapping communities in the 3.2–5.6 μm and 5.6–10 μm ranges in phase 2. The 1.8–3.2 μm size range in phase 1 and the 1–1.8 μm size range in phase 2 were grouped, indicating similar population structures.

#### 3.2.3 Size-resolved diversity of insects and nematodes pathogens and human pathogens

**Figure 4:**
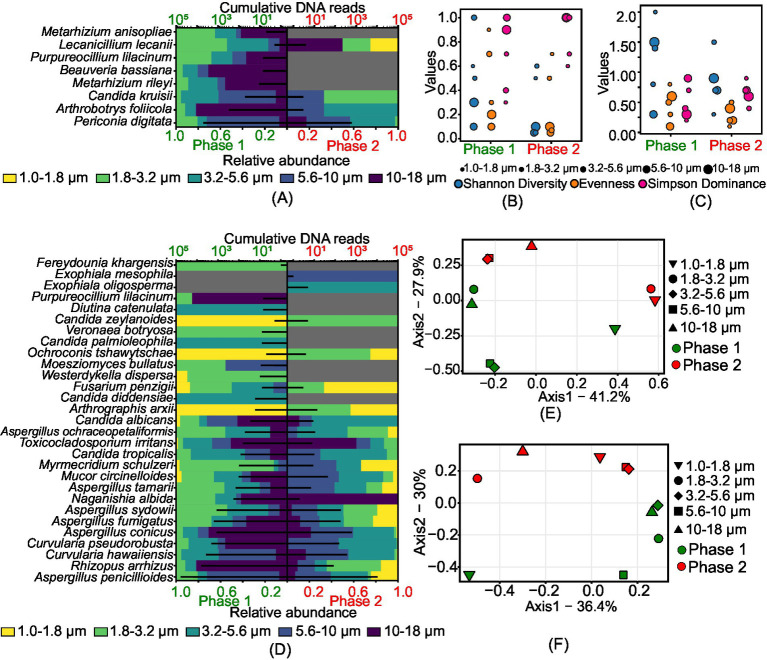
Size-resolved diversity of species identified as insects and nematodes and human pathogens during phase 1 and phase 2: **(A)** cumulative DNA sequences and the size-resolved relative abundance of insects and nematodes pathogens; **(B)** size-resolved diversity of insects and nematodes pathogens explained through the various diversity indices like Shannon diversity index, evenness, and Simpson’s dominance; **(C)** size-resolved diversity of human pathogens explained through the various diversity indices like Shannon diversity index, evenness, and Simpson’s dominance; **(D)** cumulative DNA sequences and the size-resolved relative abundance of human pathogens; **(E)** inter-size range diversity of insects and nematodes pathogens; and **(F)** inter-size range diversity of human pathogens.

A greater diversity of insects and nematodes pathogens was observed in phase 1 compared to phase 2. Figures 4A,B,E shows the size-resolved diversity of insects and nematode pathogens. Specifically, the size ranges of 1.8–3.2 μm and 3.2–5.6 μm in phase 1 had more species, with 7 and 6 species, respectively. In phase 2, the size range of 5.6–10 μm had the most species, with 3. Diversity was higher in all size ranges during Phase 1, while phase 2 had lower diversity. Evenness values, which indicate how evenly species are distributed, were higher in Phase 1 (0.1 to 0.9) compared to phase 2 (0.05 to 0.7). Dominance, or the presence of a few species being very common, was higher in phase 2, reaching a maximum value of 1. Principal Coordinate Analysis (*PCoA*) showed distinct population structures in both phases, except for the size ranges of 3.2–5.6 μm and 5.6–10 μm, which had overlapping communities. This indicates that phase 1 had a higher diversity of size-specific species than phase 2, despite some overlap in certain size ranges.

Figures 4D,C,F illustrates the type and size-resolved diversity of human pathogens. *Aspergillus penicillioides* was common in both phases. Unique to phase 1 were species like *Candida diddensiae*, *Candida palmioleophila*, *Diutina catenulata*, *Purpureocillium lilacinum*, *Veronaea botryosa*, and *Westerdykella dispersa*, while *Exophiala mesophila* and *Exophiala oligosperma* were specific to phase 2. The size range of 1.8–3.2 μm had the highest number of species in both phases, with a total of 21 species in phase 1 and 16 in phase 2. In phase 1, the Shannon diversity index (*H*) ranged from 0.3 to 2, evenness from 0.1 to 0.8, and dominance (*D*) from 0.2 to 0.9. In phase 2, the diversity index (*H*) ranged from 0.3 to 1.5, evenness from 0.1 to 0.5, and dominance (*D*) from 0.4 to 0.9. Principal Coordinates Analysis (*PCoA*) showed highly diverse communities across all size ranges in both phases, except for overlapping structures in the 3.2–5.6 μm and 5.6–10 μm ranges in phase 2. This analysis indicates that phase 1 had a higher diversity of size-specific species than phase 2.

#### 3.2.4 Size-resolved diversity of beneficial/useful fungi

**Figure 5:**
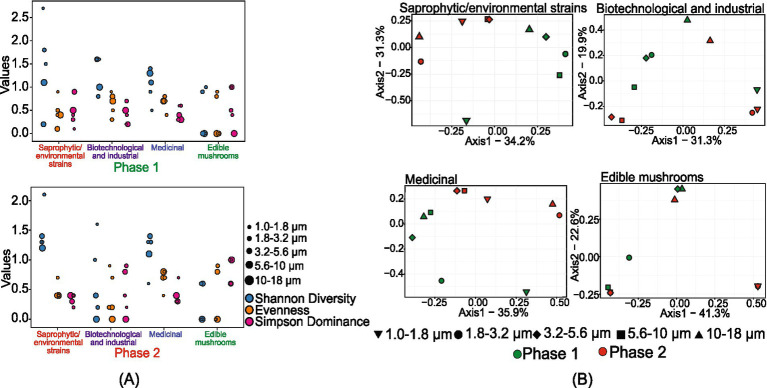
Size-resolved diversity of species identified as beneficial/useful species during phase 1 and phase 2: **(A)** size-resolved diversity explained through the various diversity indices like Shannon diversity index, evenness, and Simpson’s dominance; and **(B)** inter-size range diversity.

The study identified 176 beneficial fungal species, including 77 saprophytic/environmental strains, 20 biotechnological and industrial strains, 8 medicinal fungi, and 5 edible mushrooms (Supplementary Figure S1). Among the saprophytic/environmental strains, *Aspergillus penicillioides* was the most dominant species in both sampling phases, followed by *Coprinopsis laani* in phase 1 and *Tilletiopsis washingtonensis* in phase 2. Figure 5 represents the size-resolved diversity of beneficial/useful fungi. In phase 1, the size ranges of 1.8–3.2 μm and 3.2–5.6 μm had the highest number of species, with 48 and 41 OTUs, respectively. In phase 2, the 1.0–1.8 μm range contained the most species (29), followed closely by the 1.8–3.2 μm and 3.2–5.6 μm ranges, each with 27 species. This indicates a shift in the distribution of species across different size ranges between the two phases.

Shannon diversity analysis further highlighted these variations. In phase 1, the 1.0–1.8 μm size range showed a highly diverse population with a 𝐻 value of 2.7. In phase 2, the highest diversity was observed in the 1.8–3.2 μm size range, with a 𝐻 value of 2.1. The diversity indices suggested that both phases exhibited high evenness and low dominance in the 1.0–1.8 μm and 1.8–3.2 μm size ranges, respectively. The unique population diversity within each size range showed distinct community structures without significant overlap, except in the 3.2–5.6 μm and 5.6–10 μm size ranges of phase 2.

For biotechnological and industrial fungi, phase 1 exhibited a high number of species in the 1.8–3.2 μm and 3.2–5.6 μm size ranges. In contrast, in phase 2, the 1.0–1.8 μm size range showed the highest number of species. Some species were uniquely observed in phase 1, including *Beauveria bassiana*, *Kluyveromyces lactis*, *Metarhizium rileyi*, *Myceliophthora thermophila*, *Pichia kluyveri*, *Pichia membranifaciens, Pseudozyma hubeiensis*, *Thermomyces dupontii*, *Trichoderma reesei*, and *Virgaria nigra*. The Shannon diversity index provided further insights into the fungal diversity observed in the samples. Phase 1 exhibited higher diversity compared to phase 2, with a maximum 𝐻 value of 1.6 at the size ranges of 1.0–1.8 μm, 1.8–3.2 μm, and 5.6–10 μm. In phase 2, the 1.0–1.8 μm size range also showed a maximum *H* value of 1.6. This higher diversity in phase 1 suggests a more varied fungal community during this period. Additionally, the maximum evenness value of 0.9 was observed in the 1.0–1.8 μm size range in both phases, indicating a well-distributed fungal population within these size ranges. High intercommunity diversity among the different size ranges in both phases indicated distinct and separate fungal communities within each size range, except for the 1.0–1.8 μm size range in phase 1 and the 1.0–1.8 μm and 1.8–3.2 μm size ranges in phase 2, which grouped together. This grouping suggests a nearly similar fungal community structure within these specific size ranges.

The size-fractioned assessment of medicinal fungi showed that the 1.8–3.2 μm size range in phase 1 samples contained the highest number of species. In phase 2, 7 species were observed in the 3.2–5.6 μm and 5.6–10 μm size ranges. The diversity analysis indicated that phase 2 exhibited higher intra-community diversity than phase 1 samples. The evenness of fungal distribution in both phases was similar, except for the 1.0–1.8 μm and 1.8–3.2 μm size ranges. The dominance (*D*) value was comparatively higher at the 1.0–1.8 μm size range in phase 2, with a value of 0.7. In phase 1, a dominance (*D*) of 0.6 was observed in the 1.0–1.8 μm and 1.8–3.2 μm size ranges, consistent with the observed diversity values.

The size-fractioned characterization of edible mushrooms indicated no observable sequences in the 10–18 μm size range in phase 1. The diversity analysis showed the dominance of a single species across various size ranges in both phases. Furthermore, the analysis suggested that the 1.8–3.2 μm size range of phase 1 exhibited a diverse community structure distinct from other size ranges. However, due to the limited number of OTUs observed in phase 2, the diversity characteristics for this phase cannot be reliably assessed as an accurate representation of the broader fungal community.

## 4 Discussion

Changes in the cell size and shape of microorganisms as they adapt to environmental conditions significantly impact the presence of fungal aerobiomes across a wide size range. We characterized fungal bioaerosols using SEM imaging and discovered a diverse range of fungal propagules within the 3–20 μm size range. These propagules include spores, fungal fragments, clusters of spores, mycelium, and spore-dust agglomerates (Krishnamoorthy et al., 2020; Lacey and Goodfellow, 1975; Tong and Bruce Lighthart, 2000). Our study also revealed that fungal OTUs was present in various size ranges varying from 1 to 18 μm, with the highest diversity observed in the 1–1.8 μm size range. This increased diversity in smaller size fractions may be attributed to the presence of smaller basidiospores in the fungal bioaerosols samples from the agricultural field.

We identified several crop pathogens that can cause epiphytic or endophytic infections, including foliar diseases, powdery mildew, cankers, and more. Presence of pathogens like *Aspergillus flavus* and *Aspergillus niger* indicate a strong probability of postharvest infections and other diseases in pulses. The presence of *Uromyces viciae-fabae* and *Puccinia recondita*, *Basidiomycota* members, suggests a risk of faba-bean rust and wheat rust, respectively, in the sampling site or nearby locations (Varghese et al., 2024). *Rhizopus arrhizus* is known for causing barn rot in tobacco (Chen et al., 2020). *Flammulina velutipes* can affect mulberry, Chinese hackberries, and persimmon trees (Fischer and Garcia, 2015). *Choanephora cucurbitarum* can cause fruit and blossom rot in cucurbits, infect okra, and cause rot in teasel gourd. *Penicillium citrinum* and *Colletotrichum capsici* – spices fungi – suggest potential opportunistic infections that could reduce yield (Ragavendran et al., 2019; Saxena et al., 2016). The literature review also revealed that the same fungal pathogens can affect multiple crops. For example, *Aspergillus flavus* can infect both cereals and pulses, while *Aspergillus niger* targets pulses, fruits, and vegetables. Other species, such as *Macrophomina phaseolina* and *Penicillium citrinum*, were found to infect multiple crop types, including cereals, pulses, cash crops, and spices. Similar observations were made by Couch et al. (2005) regarding the ability of *Magnaporthe oryzae* to infect multiple crops.

The presence of certain pathogenic fungal species indicated that the local plant species in the sampling site were susceptible to a variety of fungal infections, including white rot (Slippers et al., 2003), wood rot, leaf spots (Limtong et al., 2020), blight, melting out, root rot (Carlucci et al., 2012), rotten trunks and leaves (Novaković et al., 2018), stem and leaf rot (Alfenas et al., 2018; Pornsuriya et al., 2017), leaf blight (Saxena et al., 2016), smut disease (Kellner et al., 2011), powdery mildew (Cowger and Brown, 2019), jelly rot (Yeo et al., 2008), cankers, fruit rot, collar rot (Rivedal et al., 2020), leaf rust, damping off, wire stem, general plant disease, leaf disease, and opportunistic infections (Okolo et al., 2015; Stoll et al., 2005). These findings were consistent with previous studies by Anonymous (2017), Fisher et al. (2012), Savary et al. (2012), and Simion (2018), which reported that the phytopathogenic fungi significantly reduce global crop yields, contaminate livestock feed, and cause various plant infections.

Several fungal pathogens identified could play a crucial role in controlling insect pests, including Culex mosquitoes, nematodes, and malarial mosquitoes (Castillo Lopez et al., 2014; Davies et al., 2015; Jiang et al., 2019; Khan et al., 2012; McKinnon et al., 2018; Pedrini et al., 2013; Ragavendran et al., 2019). As highlighted by Brown et al. (2012), Fisher et al. (2012, 2018), and Rhodes (2019), certain fungal pathogens are capable of causing severe diseases, particularly in immunocompromised individuals. The potential health impacts are wide-ranging and include opportunistic infections (Bezerra et al., 2017; Rudramurthy et al., 2019), neonatal sepsis (Okolo et al., 2015), and nosocomial infections such as candidiasis (Kim and Singh, 2000). Additional severe health issues identified include candidemia, infections associated with intravenous catheters (Yamin et al., 2021), Hickman catheter-associated fungemia (Whitby et al., 1996), allergies (Gunasekaran, 2017), and infections in immunocompromised individuals (Benedict and Mody, 2016). Other conditions include nail infections, infections in patients with ketoacidosis, cutaneous lesions, and more severe conditions such as golden tongue, infections in transplant patients, and angio-invasive infections. These fungal pathogens also pose a risk to animals, which can, in turn, enhance the likelihood of human infections and potential epidemic outbreaks (Gnat et al., 2021; Köhler et al., 2015; WHO, 2018). The results of the size-resolved diversity analysis aligns with the findings of Fröhlich-Nowoisky et al. (2016), Guarnieri and Balmes (2014), Hofmann (2011), and Nazaroff (2016) who noted that human pathogenic fungal bioaerosols are predominantly within the size varying from 1.8 to 10 μm. The size varying from 1.8 to 5.6 μm are particularly significant for the diverse community structure of *human pathogens* (Guarnieri and Balmes, 2014; Krishnamoorthy et al., 2020).

The *saprophytic/environmental* strains included a diverse array of species, including aquatic fungi (Jooste et al., 1990), marine fungi (Wang et al., 2017), wood-decaying fungi, xerotolerant fungi (Hirooka et al., 2016), soil fungi, environmental yeasts (Li et al., 2021), rare environmental mushrooms, fungi that thrive on minerals and mineral-rich rocks (Jiang et al., 2018), extremotolerant fungi, weeping widow mushrooms (Roberts and Evans, 2011), and dung fungi and mushrooms. These environmental fungi and saprophytes are ubiquitous and play crucial roles in maintaining the carbon-nitrogen cycle, the decomposition of organic matter, and various other ecological processes and cycles (Dagenais and Keller, 2009). The unique population diversity within each size range, showing distinct community structures without significant overlap, except in the 3.2–5.6 μm and 5.6–10 μm size ranges of phase 2, suggests a less distinct separation of fungal communities in these specific size ranges during Phase 2, highlighting the diverse nature of *saprophytic/environmental* strains present in the environment.

Among the *Biotechnologically and industrially important* fungi, *Penicillium polonicum* stood out due to its extensive applications in producing penicillic acid, verrucosidin, patulin, anacine, 3-methoxyviridicatin, and glycopeptides (Valente et al., 2021). Penicillic acid is known for its antibiotic properties, while verrucosidin and patulin are mycotoxins with potential uses in biocontrol and pharmaceuticals. Anacine is an alkaloid with potential therapeutic applications, and 3-methoxyviridicatin and glycopeptides are compounds with antimicrobial properties that can be used in medicine and agriculture. *Beauveria bassiana* is known for its use as a bio-pesticide due to its pathogenicity against a wide range of insect pests. *Kluyveromyces lactis* is utilized in the dairy industry for the production of lactose-free milk and other dairy products. *Metarhizium rileyi* is another important bio-pesticide, especially effective against caterpillar pests. *Myceliophthora thermophila* and *Thermomyces dupontii* are thermophilic fungi used in the production of industrial enzymes that can withstand high temperatures, making them valuable in various industrial processes. *Pichia kluyveri* and *Pichia membranifaciens* are known for their roles in fermentation, particularly in the production of bioethanol and other biofuels. *Trichoderma reesei* is widely used in the production of cellulases and hemicellulases, enzymes essential for the biofuel industry. *Virgaria nigra*, although less well-known, has potential applications in bioremediation and the production of secondary metabolites with pharmaceutical properties.

In the case of *medicinal fungi*, *Lenzites betulina*, known for its anticancer and antimicrobial properties (Liu et al., 2014), was uniquely observed in phase 1. In contrast, *Ganoderma lucidum*, which has various medicinal benefits, including stabilizing blood glucose levels, modulating the immune system, providing hepatoprotection, and exhibiting bacteriostatic properties (Unlu et al., 2016), was specifically observed in phase 2. Among the *medicinal fungi* identified in the bioaerosols, *Trametes versicolor* stood out as a particularly potent strain due to its extensive medical and immunological applications. This species is known for its ability to activate the reticuloendothelial system, modulate cytokine production (specifically enhancing INF and IL-2), improve the viability of dendritic cells, promote T-cell maturation, enhance the activity of natural killer cells, and stimulate antibody production (Knežević et al., 2018). Furthermore, *Trametes versicolor* has demonstrated antitumor and anticancer effects, making it a significant finding in this study. Size-resolved diversity analysis revealed that the communities present in all size ranges exhibited unique, non-overlapping populations in *medicinal fungi*. This suggests that the *medicinal fungi* observed in the bioaerosols had a more diverse community structure than other functional categories of fungal bioaerosols.

## 5 Conclusion

In conclusion, this study provides the first comprehensive, size-resolved characterization of fungal bioaerosols over an agricultural ecosystem in India during the winter season, highlighting clear seasonal and size-dependent variations in diversity, abundance, and ecological function. The observed enrichment of fungal diversity in smaller particle size ranges (1–1.8 μm) suggests that fine fungal spores contribute significantly to atmospheric biodiversity and may influence both local and regional climate - ecosystem interactions. Moreover, the coexistence of pathogenic and beneficial fungal taxa across size fractions underscores the complex interplay between agricultural practices, environmental stressors, and fungal adaptation mechanisms driven by ongoing climatic changes.

The identification of multiple crop pathogens as well as human pathogenic fungal species concentrated in the 1.8–3.2 μm size range, signals a potential risk of airborne transmission and long-range dispersal across ecosystems. Such findings emphasize the urgent need for continuous, high-resolution bioaerosol monitoring and integrative surveillance strategies to mitigate the threats posed by fungal pathogens to both crop productivity and public health. Establishing systematic, size-resolved bioaerosol databases and linking them with climatic and genomic data will be critical for developing predictive models and effective policy frameworks aimed at safeguarding ecosystem stability and food security in a changing environment.

## Data Availability

The datasets presented in this study can be found in online repositories. The names of the repository/repositories and accession number(s) can be found below: https://www.ncbi.nlm.nih.gov/, SRR22012342, SRR22012343, SRR22012344, SRR22012345, SRR22012346, SRR22012347, SRR22012348, SRR22012349, SRR22012350, SRR22012351.
