# Supplementary material for: Size-resolved fungal bioaerosol diversity over an Indian agricultural field and their ecosystem-health implications
Source: Front Microbiol. 2025 Dec 3;16:1648820. doi: 10.3389/fmicb.2025.1648820 (PMC12708607; doi:10.3389/fmicb.2025.1648820)
Supplement: Supplementary file 1 [file Table_1.DOCX]

**Supplementary Table S1**: Brief description of properties of the fungal OTUs identified with pathogenic potential

| **Fungal species** | **Crop pathogens** | | | | | | **Plant** | **Insect and nematodes** | **Human** | **Reference** |
| --- | --- | --- | --- | --- | --- | --- | --- | --- | --- | --- |
|  | **Cereals** | **Pulses** | **Cash crops** | **Fruits** | **Vegetables** | **Spices** |  |  |  |  |
| *Aspergillus flavus* | Affects grains, crops, and causes post-harvest storage diseases | post-harvest storage diseases | - | - | - | - | - | - | Opportunistic pathogen causes aspergillosis | (Atongbiik Achaglinkame, Opoku, and Amagloh 2017; El-Shanshoury et al. 2014; Rudramurthy et al. 2019) |
| *Aspergillus halophilicus* | Post-harvest storage disease, especially in dried corns | - | - | - | - | - | - | - | - | (Christensen, Papavizas, and Benjamin 1959) |
| *Bipolaris melinidis* | leaf spots, blights, melting out, and root rot of paddy, maize, wheat, and sorghum | - | - | - | - | - | - | - | - | (Manamgoda et al. 2014) |
| *Curvularia intermedia* | Affects crops especially paddy and sorghum | - | - | - | - | - | - | - | - | (Li et al. 2019) |
| *Curvularia lunata* | seed blight and germination failure in paddy, wheat | Seed blight and germination failure of millets | - | - | - | - | Leaf spots in flowering plants | - | - | (Akram et al. 2014; Limtong, Into, and Attarat 2020) |
| *Erysiphe polygoni* | Powdery mildew of buckwheat | - | - | - | - | - | - | - | - | (Lu et al. 2015) |
| *Kabatiella zeae* | Leaf spot and stalk rot in maize | - | - | - | - | - | - | - | - | (Pronczuk, Bojanowski, and Warzecha 2004) |
| *Macrophomina phaseolina* | Damping off, seedling blight, collar, basal stem, charcoal, root rot of sorghum, wheat, corn, and alpha alpha | Root rot of chickpea, soyabean | Root rot of peanuts, sunflower, sesame seeds | - | Root rot of cabbage, sweet potato, and potato | - | - | - | - | (Egel et al. 2020; Su et al. 2001; Ullah et al. 2019) |
| *Moesziomyces bullatus* | - | Millet smut | - | - | - | - | pathogenic | - | Neonatal sepsis | (Okolo et al. 2015; Stoll, Begerow, and Oberwinkler 2005) |
| *Nigrospora oryzae* | Grain spots in paddy, sorghum, and corn | - | Leaf blight and spots in cotton and tea | - | - | - | - | - | - | (L. M. Liu et al. 2021; Zhang et al. 2012) |
| *Penicillium citrinum* | Pathogenic | - | - | Pathogenic | - | Pathogenic | - | Culex mosquito mortality | - | (Carroll 1986; Davies et al. 2021; Ragavendran et al. 2019) |
| *Penicillium polonicum* | Spoilage cereals | - | Spoilage of peanuts | Spoilage of citrus fruits | Spoilage of onions | - | - | - | - | (Çakır and Maden 2015; Duduk, Vasić, and Vico 2014; NÚÑEZ et al. 2000) |
| *Puccinia recondita* | Leaf rust in wheat and rey | - | - | - | - | - | - | - | - | (Peksa and Bankina 2019) |
| *Rhodosporidiobolus nylandii* | Affects leaves of corn | - | - | - | - | - | Leaf pathogen | - | - | (Satianpakiranakorn, Khunnamwong, and Limtong 2020) |
| *Sporisorium lepturi* | Smut especially in sorghum | - | - | - | - | - | Smut disease | - | - | (Kellner et al. 2011) |
| *Sporisorium reilianum* | Pathogen of maize and sorghum affects inflorescence | - | - | - | - | - | - | - | - | (Poloni and Schirawski 2016) |
| *Tilletia barclayana* | Pathogen of paddy causes black bust with smutted appearance | Infects Pearl millets | - | - | - | - | Infects signal grass and crab grass | - | - | (Babadoost and Mathre 1998; Elshafey 2018) |
| *Ustilaginoidea virens* | Smut of paddy crops | - | - | - | - | - | - | - | - | (Jiehua et al. 2019) |
| *Ustilago maydis* | Smut of corn and maize | - | - | - | - | - | - | - | - | (Lanver et al. 2017) |
| *Zymoseptoria brevis* | Leaf disease of barley | - | - | - | - | - | - | - | - | (Quaedvlieg et al. 2011) |
| *Blumeria graminis* | Powdery mildew of cereals | - | - | - | - | - | Powdery mildew in grass | - | - | (Cowger and Brown 2019) |
| *Aspergillus niger* | - | Black mold disease commonly observed in pulses | Black mold disease of peanuts | Black mold disease of grapes, apricots, etc. | Black mold disease especially onions | - | - | - | - | (Bianchini and Stratton 2014; Erkmen and Bozoglu 2016) |
| *Choanephora cucurbitarum* | - | Rot of snap bean and southern pea, stem and leaf rot of hyacinth bean and green pea | - | - | Fruit and blossom rot of cucurbits and affects okra | - | Stem and leaf rot of *Withania somnifera* (ashwagandha), and teasle guard | - | - | (Alfenas et al. 2018; Pornsuriya et al. 2017; Saroj et al. 2012) |
| *Colletotrichum capsici* | - | Leaf blight of chickpea, dieback in pigeon pea | - | - | Leaf blight in peppers like chilly and capsicum | Affects pepper | Leaf blight in *Chlorophytum borivilianum*, and basil, anthracnose in poinsettia | - | - | (Montri, Taylor, and Mongkolporn 2009; Saxena et al. 2016) |
| *Uromyces viciae-fabae* | - | Causes faba-bean rust | - | - | - | - | - | - | - | (Conner and Bernier 1982; Graham and Vance 2003; Voegele 2006) |
| *Pestalotiopsis coffeae-arabicae* | - | - | Found on the leaf of *Coffee arabica* and opportunistic pathogen capable of producing chemically novel metabolites | - | - | - | - | - | - | (SONG et al. 2013) |
| *Alternaria longissima* | - | - | Causes leaf spot, foliage blight, stem necrosis and spot of Sesamum | - | - | - | - | - | - | (Naik et al. 2017) |
| *Rhizopus arrhizus* | - | - | Causes barn rot of tobacco | - | - | - | - | - | - | (Chen et al. 2020; Kortekamp, Schmidtke, and Serr 2003) |
| *Aplosporella javeedii* | - | - | - | Causes branch blight disease in mulberries | - | - | - | - | - | (Jia et al. 2019) |
| *Aspergillus carbonarius* | - | - | - | Affects grape fruits | - | - | - | - | - | (C. Jiang, Shi, and Zhu 2013) |
| *Aureobasidium pullulans* | - | - | - | Epiphyte and endophyte of apple and grapes | Epiphyte and endophyte of cucumber, green beans and cabbage | - | - | - | - | (Barata, Malfeito-Ferreira, and Loureiro 2012; Heidenreich et al. 1997; Pinto et al. 2018; Zajc et al. 2020) |
| *Candida hyderabadensis* | - | - | - | A beneficial fungus observed in association with grapes and an opportunistic pathogen | - | - | - | - | - | (Rao et al. 2007) |
| *Dothiorella vinea-gemmae* | - | - | - | Associated with grapes an opportunistic pathogen | - | - | - | - | - | (Pitt, Úrbez-Torres, and Trouillas 2015) |
| *Eutypa lata* | - | - | - | Wood rot of grape plant leading to dead arm and grape cankers | - | - | - | - | - | (Catal et al. 2007; Rolshausen et al. 2014) |
| *Flammulina velutipes* | - | - | - | Opportunistic pathogen of Chinese hackberry trees, ash plant, mulberry, and persimmon trees | - | - | - | - | - | (Fischer and Garcia 2015) |
| *Hanseniaspora uvarum* | - | - | - | Observed in wine making environments and opportunistic pathogen | - | - | - | - | - | (Albertin et al. 2016) |
| *Penicillium aurantiogriseum* | - | - | - | Infects strawberry significant loss observed during post-harvest period | - | - | Infects asparagus | - | - | (Bouhoudan, Chidi, and Khaddor 2020; Moslem et al. 2010) |
| *Pichia kluyveri* | - | - | - | Helps in wine making and improves wine quality, could act as an opportunistic pathogen | - | - | - | - | - | (Méndez-Zamora et al. 2020) |
| *Pichia membranifaciens* | - | - | - | Opportunistic pathogen of fruits | - | - | - | - | - | (WANG et al. 2018) |
| *Plectosphaerella cucumerina* | - | - | - | Causes fruit rots | - | - | Causes root and collar rots | - | - | (Carlucci et al. 2012; Rivedal et al. 2020; Slippers et al. 2003; Xu et al. 2014) |
| *Amylostereum laevigatum* | - | - | - | - | - | - | Plant pathogen causes white rot on trees | - | - | (Slippers et al. 2003) |
| *Antrodiella brasiliensis* | - | - | - | - | - | - | Plant pathogen causes crust like wood rot | - | - | Westphalen et al., 2019 |
| *Candida boleticola* | - | - | - | - | - | - | Plant pathogen | - | - | (Kurtzman et al. 2015) |
| *Coprinellus disseminatus* | - | - | - | - | - | - | Plant pathogen grows on rotting trees | - | - | (Novaković et al. 2018) |
| *Cylindrobasidium evolvens* | - | - | - | - | - | - | Plant pathogen grows on dead branches of deciduous trees | - | - | (Burneviča et al. 2016) |
| *Daedaleopsis confragosa* | - | - | - | - | - | - | Plant pathogen causes white rot of willow trees | - | - | (Ćilerdžić et al. 2019) |
| *Entyloma diastateae* | - | - | - | - | - | - | Smut fungi causes leaf spots in plants | - | - | (Rooney-Latham et al. 2017) |
| *Erysiphe multappendicis* | - | - | - | - | - | - | Causes powdery mildew of plants | - | - | (Abasova, Aghayeva, and Takamatsu 2018) |
| *Macalpinomyces ewartii* | - | - | - | - | - | - | Causes smut disease of plants | - | - | (McTaggart et al. 2012) |
| *Meripilus giganteus* | - | - | - | - | - | - | Polyporous white rot pathogen especially broad leaf tress like Abies, *Picea, Pinus, Quercus* and *Ulmus* species | - | - | (Y. S. Kim and Singh 2000; SCHWARZE and FINK 1998) |
| *Microbotryum cordae* | - | - | - | - | - | - | Common plant pathogen | - | - | (Spooner and N.W. legon 2006) |
| *Mycosphaerella ellipsoidea* | - | - | - | - | - | - | Causes leaf disease of *Eucalyptus globulus* | - | - | (Hunter et al. 2004) |
| *Mycosphaerella tassiana* | - | - | - | - | - | - | Infects several plant hosts | - | - | (Petrie and Vanterpool 1978) |
| *Phlebia tremellosa* | - | - | - | - | - | - | Plant pathogen commonly known as trembling Merulius or jelly rot a wood decaying fungus found in rotting hard wood and conifer plants | - | - | (Vares, Niemenmaa, and Hatakka 1994; Yeo, Kim, and Choi 2008) |
| *Pholiota highlandensis* | - | - | - | - | - | - | Plant pathogen which grows in clusters in the charred base of trees | - | - | (Matheny et al. 2018) |
| *Phoma herbarum* | - | - | - | - | - | - | Causes brown leaf spots and cankers | - | - | (Thangaraj et al. 2018) |
| *Phyllosticta capitalensis* | - | - | - | - | - | - | Endophytic fungi cause leaf spots of ornamental plants | - | - | (Cheng et al. 2019; Liao et al. 2020) |
| *Pisolithus albus* | - | - | - | - | - | - | Plant pathogen Tunisia and *Eucalyptus occidentalis* | - | - | (Jaouani et al. 2015) |
| *Sarocladium glaucum* | - | - | - | - | - | - | Common plant pathogen | - | - | (Giraldo et al. 2015) |
| *Steccherinum ochraceum* | - | - | - | - | - | - | Plant pathogenic polyporous wood rotting fungi | - | - | (Moiseenko et al. 2019, 2020) |
| *Stereum rugosum* | - | - | - | - | - | - | Plant pathogenic polyporous wood rotting fungi, otherwise known as leaf fungus, wax fungus, and shelf fungus | - | - | (Mirić and Stefanović 2018; Vabeikhokhei et al. 2019) |
| *Thanatephorus cucumeris* | - | - | - | - | - | - | Plant pathogen with a wide host range and worldwide distribution. Further, cause various plant diseases such as collar rot, root rot, damping off, and wire stem | - | - | (Elliott et al. 2008) |
| *Toxicocladosporium irritans* | - | - | - | - | - | - | Common plant pathogen | - | Opportunistic pathogen | (Bezerra et al. 2017; Crous et al. 2007) |
| *Trametes hirsuta* | - | - | - | - | - | - | Plant pathogen known as hairy bracket fungi causes white rot of wood | - | - | (Patil and Yadav 2018; Vasina et al. 2017) |
| *Drechslera catenaria* | - | - | - | - | - | - | Pathogen causing leaf blight and brown rot in Toronto creeping bentgrass | - | - | (Larsen et al. 1981; Spilker and Larsen 1985) |
| *Arthrographis arxii* | - | - | - | - | - | - | - | - | Common pathogen | (Giraldo et al. 2014) |
| *Aspergillus conicus* | - | - | - | - | - | - | - | - | Xerophilic species causes infection in human | (Smith et al. 2013) |
| *Aspergillus fumigatus* | - | - | - | - | - | - | - | - | Causes disease in immunodeficient person | (Latgé 1999) |
| *Aspergillus ochraceopetaliformis* | - | - | - | - | - | - | - | - | Causes nail infection | (Brasch et al. 2009) |
| *Aspergillus penicillioides* | - | - | - | - | - | - | - | - | Causes infection | (Samson and Lustgraaf 1978) |
| *Aspergillus sydowii* | - | - | - | - | - | - | - | - | Occasional Human pathogen | (Rodriguez-Palacios et al. 2016; Soler-Hurtado et al. 2016) |
| *Aspergillus tamarii* | - | - | - | - | - | - | - | - | Causes pathogenic infection | (Homa et al. 2019) |
| *Candida albicans* | - | - | - | - | - | - | - | - | Common human pathogen well known for nosocomial infection | (J. Kim and Sudbery 2011) |
| *Candida diddensiae* | - | - | - | - | - | - | - | - | Reported to cause nosocomial | (S. E. Kim et al. 2020) |
| *Candida palmioleophila* | - | - | - | - | - | - | - | - | Opportunistic pathogen reported to cause intravenous catheter associated fungemia | (Jensen and Arendrup 2011; W.-L. Liu et al. 2019; Yamin, Husin, and Harun 2021) |
| *Candida tropicalis* | - | - | - | - | - | - | - | - | Causes pathogenic infection | (Chai, Denning, and Warn 2010) |
| *Candida zeylanoides* | - | - | - | - | - | - | - | - | Opportunistic pathogen causes Hickman catheter associated fungemia | (Hazen 1995; Whitby, Madu, and Bronze 1996) |
| *Curvularia hawaiiensis* | - | - | - | - | - | - | - | - | Causes pathogenic infection and allergy | (Dr Santhi Gunasekaran et al. 2017; Rinaldi et al. 1987) |
| *Curvularia pseudorobusta* | - | - | - | - | - | - | - | - | Causes pathogenic infection and allergy | (Marin-Felix, Hernández-Restrepo, and Crous 2020) |
| *Diutina catenulata* | - | - | - | - | - | - | - | - | Causes superficial to invasive infection in human and animals | (O’Brien et al. 2018) |
| *Fereydounia khargensis* | - | - | - | - | - | - | - | - | Opportunistic pathogenic yeast causing disease in immunocompromised and immunosuppressive patients | (Benedict and Mody 2016; WHO 2018) |
| *Fusarium penzigii* | - | - | - | - | - | - | - | - | Acts as agents of trauma-related eye infections of humans | (do Carmo et al. 2016; Latenser 2003) |
| *Mucor circinelloides* | - | - | - | - | - | - | - | - | Causes infection to animals like cattle and swine, occasionally causes cutaneous infection to human and Ketoacidosis patients are particularly at risk | (López-Fernández et al. 2018; Vellanki et al. 2020) |
| *Myrmecridium schulzeri* | - | - | - | - | - | - | - | - | Acts as a contaminant of bronchoscopy fluid and causes Golden Tongue syndrome | (Rippon et al. 1985) |
| *Naganishia albida* | - | - | - | - | - | - | - | - | Causes cutaneous lesions | ( Aghaei Gharehbolagh et al. 2017) |
| *Ochroconis tshawytschae* | - | - | - | - | - | - | - | - | Causes subcutaneous Phaeohyphomycosis | (Ge et al. 2012) |
| *Purpureocillium lilacinum* | - | - | - | - | - | - | - | Insect pathogen, has antinematode activity controls the growth of root knot nematodes | Causes pulmonary disease in human | (Castillo Lopez et al. 2014; Khan et al. 2012; Singh, Pandey, and Goswami 2013) |
| *Veronaea botryosa* | - | - | - | - | - | - | - | - | Causes infectious complications in transplant patients | (Welfringer et al. 2017) |
| *Westerdykella dispersa* | - | - | - | - | - | - | - | - | Causes Angio invasive infection in immunocompromised patients | (Benedict and Mody 2016; WHO 2018) |
| *Exophiala mesophila* | - | - | - | - | - | - | - | - | Causes disease in immunocompetent and immunocompromised persons | (Benedict and Mody 2016; WHO 2018) |
| *Exophiala oligosperma* | - | - | - | - | - | - | - | - | Causes human infection predominantly in immunocompromised hosts | (Benedict and Mody 2016; WHO 2018) |
| *Beauveria bassiana* | - | - | - | - | - | - | - | Parasitic to arthropods causing white muscardine disease hence called as entomopathogenic fungi mostly used as a biological insecticide to control a number of pests such as termites, thrips, whiteflies, aphids, different beetles, bedbugs and malaria transmitting mosquitoes |  | (McKinnon et al. 2018; Pedrini et al. 2013) |
| *Candida kruisii* | - | - | - | - | - | - | - | Insect pathogen grows in the gut of the insect |  | (Suh, Nguyen, and Blackwell 2006) |
| *Lecanicillium lecanii* | - | - | - | - | - | - | - | Entomopathogenic fungus which attacks white fly and aphids |  | (Trinh, Ha, and Qiu 2020; Xie et al. 2015) |
| *Metarhizium anisopliae* | - | - | - | - | - | - | - | Insect pathogen helps in controlling malarial mosquito |  | (W. Jiang et al. 2019; Mnyone et al. 2012; Rasgon 2011) |
| *Metarhizium rileyi* | - | - | - | - | - | - | - | Entomopathogenic fungi used as biopesticide |  | (Fronza et al. 2017; Visalakshi et al. 2020) |
| *Arthrobotrys foliicola* | - | - | - | - | - | - | - | Nematode pathogen that feeds on nematode |  | (Liou and Tzean 1997; Yang et al. 2011) |
| *Periconia digitata* | - | - | - | - | - | - | - | Antinematode activity |  | (Somarathne et al. 2018; Zhu et al. 2008) |

**References**

Abasova, Lamiya V., D. N. Aghayeva, and S. Takamatsu. 2018. “Notes on Powdery Mildews of the Genus Erysiphe from Azerbaijan.” *Current Research in Environmental and Applied Mycology* 8(1): 30–53.

Aghaei Gharehbolagh, S et al. 2017. “First Case of Superficial Infection Due to Naganishia Albida (Formerly Cryptococcus Albidus) in Iran: A Review of the Literature.” *Current Medical Mycology* 3(2): 33–37. http://cmm.mazums.ac.ir/article-1-158-en.html.

Akram, W, T Anjum, A Ahmad, and R Moeen. 2014. “First Report of Curvularia Lunata Causing Leaf Spots on Sorghum Bicolor from Pakistan.” *Plant Disease* 98(7): 1007. https://doi.org/10.1094/PDIS-12-13-1291-PDN.

Albertin, Warren et al. 2016. “Hanseniaspora Uvarum from Winemaking Environments Show Spatial and Temporal Genetic Clustering.” *Frontiers in Microbiology* 6(JAN).

Alfenas, R. F., S. M. Bonaldo, R. A.S. Fernandes, and M. R.N. Colares. 2018. “First Report of Choanephora Cucurbitarum on Crotalaria Spectabilis: A Highly Aggressive Pathogen Causing a Flower and Stem Blight in Brazil.” *Plant Disease* 102(7): 1456.

Atongbiik Achaglinkame, Matthew, Nelson Opoku, and Francis Kweku Amagloh. 2017. “Aflatoxin Contamination in Cereals and Legumes to Reconsider Usage as Complementary Food Ingredients for Ghanaian Infants: A Review.” *Journal of Nutrition and Intermediary Metabolism* 10: 1–7. https://doi.org/10.1016/j.jnim.2017.09.001.

Babadoost, M., and D. E. Mathre. 1998. “A Method for Extraction and Enumeration of Teliospores of Tilletia Indica , T. Controversa , and T. Barclayana in Soil.” *Plant Disease* 82(12): 1357–61. https://apsjournals.apsnet.org/doi/10.1094/PDIS.1998.82.12.1357.

Barata, A, M Malfeito-Ferreira, and V Loureiro. 2012. “The Microbial Ecology of Wine Grape Berries.” *International Journal of Food Microbiology* 153(3): 243–59. https://linkinghub.elsevier.com/retrieve/pii/S0168160511006878.

Benedict, Kaitlin, and Rajal K. Mody. 2016. “Epidemiology of Histoplasmosis Outbreaks, United States, 1938-2013.” *Emerging Infectious Diseases* 22(3): 370–78.

Bezerra, Jadson D P et al. 2017. “New Endophytic Toxicocladosporium Species from Cacti in Brazil, and Description of Neocladosporium Gen. Nov.” *IMA Fungus* 8(1): 77–97. https://doi.org/10.5598/imafungus.2017.08.01.06.

Bianchini, A, and J Stratton. 2014. “SPOILAGE OF ANIMAL PRODUCTS | Spoilage of Plant Products: Cereals and Cereal Flours.” In *Encyclopedia of Food Microbiology (Second Edition)*, eds. Carl A Batt and Mary Lou Tortorello. Oxford: Academic Press, 459–64. https://www.sciencedirect.com/science/article/pii/B9780123847300003128.

Bouhoudan, A, F Chidi, and M Khaddor. 2020. “Penicillium Aurantiogriseum : A Great Potential for Biotechnology.” (February).

Brasch, J. et al. 2009. “Nail Infection by Aspergillus Ochraceopetaliformis.” *Medical Mycology* 47(6): 658–62. https://academic.oup.com/mmy/article-lookup/doi/10.1080/13693780902803032.

Burneviča, NATĀLIJA et al. 2016. “Fungi Inhabiting Bark Stripping Wounds Made by Large Game on Stems of Picea Abies (L.) Karst. in Latvia.” *Balt For* 22: 2–7.

Çakır, E., and S. Maden. 2015. “ First Report of Penicillium Polonicum Causing Storage Rots of Onion Bulbs in Ankara Province, Turkey .” *New Disease Reports* 32(1): 24–24.

Carlucci, A., M.L. Raimondo, J. Santos, and A.J.L. Phillips. 2012. “Plectosphaerella Species Associated with Root and Collar Rots of Horticultural Crops in Southern Italy.” *Persoonia - Molecular Phylogeny and Evolution of Fungi* 28(1): 34–48. https://www.ingentaconnect.com/content/10.3767/003158512X638251.

do Carmo, Anália et al. 2016. “Fusarium Dimerum Species Complex (Fusarium Penzigii) Keratitis After Corneal Trauma.” *Mycopathologia* 181(11–12): 879–84. http://link.springer.com/10.1007/s11046-016-0060-1.

Carroll, G C. 1986. “The Biology of Endophytism in Plants with Particular Reference to Woody Perennials.” In *Microbiology of the Phyllosphere*, Cambridge University Press, 203–22.

Castillo Lopez, Diana, Keyan Zhu-Salzman, Maria Julissa Ek-Ramos, and Gregory A. Sword. 2014. “The Entomopathogenic Fungal Endophytes Purpureocillium Lilacinum (Formerly Paecilomyces Lilacinus) and Beauveria Bassiana Negatively Affect Cotton Aphid Reproduction under Both Greenhouse and Field Conditions” ed. Thomas L. Wilkinson. *PLoS ONE* 9(8): e103891. https://dx.plos.org/10.1371/journal.pone.0103891.

Catal, M, S A Jordan, S C Butterworth, and A M C Schilder. 2007. “Detection of Eutypa Lata and Eutypella Vitis in Grapevine by Nested Multiplex Polymerase Chain Reaction.” *Phytopathology®* 97(6): 737–47. https://doi.org/10.1094/PHYTO-97-6-0737.

Chai, Louis Yi Ann, David W Denning, and Peter Warn. 2010. “Candida Tropicalis in Human Disease.” *Critical Reviews in Microbiology* 36(4): 282–98. https://doi.org/10.3109/1040841X.2010.489506.

Chen, Qian Li et al. 2020. “Fungal Composition and Diversity of the Tobacco Leaf Phyllosphere During Curing of Leaves.” *Frontiers in Microbiology* 11(September).

Cheng, Lin-Lin et al. 2019. “Phyllosticta Capitalensis Causes Leaf Spot on Tea Plant (Camellia Sinensis) in China.” *Plant Disease* 103(11): 2964. https://doi.org/10.1094/PDIS-04-19-0768-PDN.

Christensen, C. M., G. C. Papavizas, and C. R. Benjamin. 1959. “A New Halophilic Species of Eurotium.” *Mycologia* 51(5): 636–40. https://www.tandfonline.com/doi/full/10.1080/00275514.1959.12024846.

Ćilerdžić, Jasmina et al. 2019. “Stimulation of Wood Degradation by Daedaleopsis Confragosa and D. Tricolor.” *Applied Biochemistry and Biotechnology* 187(4): 1371–83. http://link.springer.com/10.1007/s12010-018-2884-2.

Conner, R L, and C C Bernier. 1982. “Race Identification in Uromyces Viciae-Fabae.” *Canadian Journal of Plant Pathology* 4(2): 157–60. https://doi.org/10.1080/07060668209501318.

Cowger, Christina, and James K.M. Brown. 2019. “Durability of Quantitative Resistance in Crops: Greater Than We Know?” *Annual Review of Phytopathology* 57: 253–77.

Crous, P.W., U. Braun, K. Schubert, and J.Z. Groenewald. 2007. “Delimiting Cladosporium from Morphologically Similar Genera.” *Studies in Mycology* 58(May 2014): 33–56. https://www.ingentaconnect.com/content/10.3114/sim.2007.58.02.

Davies, Catheryn R. et al. 2021. “Evolving Challenges and Strategies for Fungal Control in the Food Supply Chain.” *Fungal Biology Reviews* 36: 15–26. https://doi.org/10.1016/j.fbr.2021.01.003.

Duduk, N, M Vasić, and I Vico. 2014. “First Report of Penicillium Polonicum Causing Blue Mold on Stored Onion (Allium Cepa) in Serbia.” *Plant Disease* 98(10): 1440. https://doi.org/10.1094/PDIS-05-14-0550-PDN.

Egel, D S, W Guan, T Creswell, and J Bonkowski. 2020. “First Report of Macrophomina Phaseolina Causing Charcoal Rot of Cucumber in Indiana.” *Plant Disease* 104(7): 2030. https://doi.org/10.1094/PDIS-11-19-2421-PDN.

El-Shanshoury, Abd El-Raheem R;, Sabha M. El-Sabbagh, Hamdy A. Emara, and Heba allah E. Saba. 2014. “Occurrence of Moulds , Toxicogenic Capability of Aspergillus Flavus and Levels of Aflatoxins in Maize , Wheat , Rice and Peanut from Markets in Central Delta Provinces , Egypt Introduction All of Us Are Concerned with the Quality.” *International Journal of Current Microbiology and Applied Sciences* 3(3): 852–65.

Elliott, P E et al. 2008. “Evaluation of Tobacco Germplasm for Seedling Resistance to Stem Rot and Target Spot Caused by Thanatephorus Cucumeris.” *Plant Disease* 92(3): 425–30. https://doi.org/10.1094/PDIS-92-3-0425.

Elshafey, Rabie A.S. 2018. “Biology of Rice Kernel Smut Disease Causal Organism Tilletia Barclayana and Its Molecular Identification.” *Journal of Phytopathology and Pest Management* 5(2): 108–28. http://www.ppmj.net/index.php/ppmj/article/view/168.

Erkmen, Osman, and T Faruk Bozoglu. 2016. *Food Microbiology, 2 Volume Set: Principles into Practice*. John Wiley & Sons.

Fischer, Michael, and Vincente Gonzalez Garcia. 2015. “An Annotated Checklist of European Basidiomycetes Related to White Rot of Grapevine (Vitis Vinifera).” *Phytopathologia Mediterranea* 54(2): 281–98. http://www.jstor.org/stable/43871836.

Fronza, Edegar, Alexandre Specht, Horacio Heinzen, and Neiva Monteiro de Barros. 2017. “Metarhizium (Nomuraea) Rileyi as Biological Control Agent.” *Biocontrol Science and Technology* 27(11): 1243–64. https://doi.org/10.1080/09583157.2017.1391175.

Ge, Yi P. et al. 2012. “First Report of Subcutaneous Phaeohyphomycosis Caused by Ochroconis Tshawytschae in an Immunocompetent Patient.” *Medical Mycology* 50(6): 637–40. https://academic.oup.com/mmy/article-lookup/doi/10.3109/13693786.2011.653834.

Giraldo, A. et al. 2014. “Phylogenetic Circumscription of Arthrographis (Eremomycetaceae, Dothideomycetes).” *Persoonia: Molecular Phylogeny and Evolution of Fungi* 32(June): 102–14.

———. 2015. “Phylogeny of <I>Sarocladium</I> (<I>Hypocreales</I>).” *Persoonia - Molecular Phylogeny and Evolution of Fungi* 34(1): 10–24. http://openurl.ingenta.com/content/xref?genre=article&issn=0031-5850&volume=34&issue=1&spage=10.

Graham, Peter H, and Carroll P Vance. 2003. “Legumes: Importance and Constraints to Greater Use.” *Plant Physiology* 131(3): 872–77. https://doi.org/10.1104/pp.017004.

Gunasekaran, Dr Santhi. 2017. “A Rare Case of Curvularia Hawaiiensis in the Ear Following Trauma.” *Journal of medical science and clinical research* 5(9): 28154–58. http://jmscr.igmpublication.org/v5-i9/131 jmscr.pdf.

Hazen, Kevin C. 1995. “New and Emerging Yeast Pathogens.” *Clinical Microbiology Reviews* 8(4): 462–78. https://journals.asm.org/doi/10.1128/CMR.8.4.462.

Heidenreich, M. C. Matteson, M. R. Corral-Garcia, E. A. Momol, and T. J. Burr. 1997. “Russet of Apple Fruit Caused by Aureobasidium Pullulans and Rhodotorula Glutinis.” *Plant Disease* 81(4): 337–42. https://apsjournals.apsnet.org/doi/10.1094/PDIS.1997.81.4.337.

Homa, Mónika et al. 2019. “Characterization of Aspergillus Tamarii Strains From Human Keratomycoses: Molecular Identification, Antifungal Susceptibility Patterns and Cyclopiazonic Acid Producing Abilities.” *Frontiers in Microbiology* 10(October): 1–11.

Hunter, Gavin C. et al. 2004. “Mycosphaerella Species Causing Leaf Disease in South African Eucalyptus Plantations.” *Mycological Research* 108(6): 672–81.

Jaouani, Atef et al. 2015. “Pisolithus Albus (Sclerodermataceae), a New Record for Tunisia.” *Flora Mediterranea* 25(January 2016): 73–78.

Jensen, Rasmus H., and Maiken C. Arendrup. 2011. “Candida Palmioleophila: Characterization of a Previously Overlooked Pathogen and Its Unique Susceptibility Profile in Comparison with Five Related Species.” *Journal of Clinical Microbiology* 49(2): 549–56. https://journals.asm.org/doi/10.1128/JCM.02071-10.

Jia, Hanqi et al. 2019. “First Report of Aplosporella Javeedii Causing Branch Blight Disease of Mulberry (Morus Alba) in China.” *Journal of Plant Diseases and Protection* 126(5): 475–77. https://doi.org/10.1007/s41348-019-00245-5.

Jiang, Chunmei, Junling Shi, and Chengyong Zhu. 2013. “Fruit Spoilage and Ochratoxin a Production by Aspergillus Carbonarius in the Berries of Different Grape Cultivars.” *Food Control* 30(1): 93–100. https://www.sciencedirect.com/science/article/pii/S0956713512004355.

Jiang, Wuji et al. 2019. “Effects of the Entomopathogenic Fungus Metarhizium Anisopliae on the Mortality and Immune Response of Locusta Migratoria.” *Insects* 11(1): 36. https://www.mdpi.com/2075-4450/11/1/36.

Jiehua, Qiu et al. 2019. “Ustilaginoidea Virens: A Fungus Infects Rice Flower and Threats World Rice Production.” *Rice Science* 26(4): 199–206.

Kellner, Ronny, Evelyn Vollmeister, Michael Feldbrügge, and Dominik Begerow. 2011. “Interspecific Sex in Grass Smuts and the Genetic Diversity of Their Pheromone-Receptor System.” *PLoS Genetics* 7(12).

Khan, Ziauddin et al. 2012. “Purpureocillium Lilacinum as a Cause of Cavitary Pulmonary Disease: A New Clinical Presentation and Observations on Atypical Morphologic Characteristics of the Isolate.” *Journal of Clinical Microbiology* 50(5): 1800–1804. https://journals.asm.org/doi/10.1128/JCM.00150-12.

Kim, Joon, and Peter Sudbery. 2011. “Candida Albicans, a Major Human Fungal Pathogen.” *The Journal of Microbiology* 49(2): 171–77. http://link.springer.com/10.1007/s12275-011-1064-7.

Kim, Seong Eun et al. 2020. “Case Report: Nosocomial Fungemia Caused by Candida Diddensiae.” *BMC Infectious Diseases* 20(1): 377. https://bmcinfectdis.biomedcentral.com/articles/10.1186/s12879-020-05095-3.

Kim, Yoon Soo, and Adya P. Singh. 2000. “MICROMORPHOLOGICAL CHARACTERISTICS OF WOOD BIODEGRADATION IN WET ENVIRONMENTS: A REVIEW.” *IAWA Journal* 21(2): 135–55. https://brill.com/view/journals/iawa/21/2/article-p135_1.xml.

Kortekamp, A, Margit Schmidtke, and Anke Serr. 2003. “Infection and Decay of Tobacco Caused by Rhizopus Oryzae / Die Infektion Und Fäulnis von Tabak Verursacht Durch Rhizopus Oryzae.” *Zeitschrift für Pflanzenkrankheiten und Pflanzenschutz / Journal of Plant Diseases and Protection* 110(6): 535–43. http://www.jstor.org/stable/43215547.

Kurtzman, Cletus P. et al. 2015. “Advances in Yeast Systematics and Phylogeny and Their Use as Predictors of Biotechnologically Important Metabolic Pathways” ed. Jens Nielsen. *FEMS Yeast Research* 15(6): fov050. https://academic.oup.com/femsyr/article-lookup/doi/10.1093/femsyr/fov050.

Lanver, Daniel et al. 2017. “Ustilago Maydis Effectors and Their Impact on Virulence.” *Nature Reviews Microbiology* 15(7): 409–21. http://dx.doi.org/10.1038/nrmicro.2017.33.

Larsen, P O, A K Hagan, B G Joyner, and D A Spilker. 1981. “Leaf Blight and Crown Rot on Creeping Bentgrass, a New Disease Caused by Drechslera Catenaria.” *Plant Disease* 65(1): 79–81.

Latenser, Barbara A. 2003. “Fusarium Infections in Burn Patients: A Case Report and Review of the Literature.” *The Journal of Burn Care & Rehabilitation* 24(5): 285–88. https://academic.oup.com/jbcr/article/24/5/285/4733751.

Latgé, Jean-Paul. 1999. “Aspergillus Fumigatus and Aspergillosis.” *Clinical Microbiology Reviews* 12(2): 310–50. https://journals.asm.org/doi/10.1128/CMR.00140-18.

Li, J, M Li, X X Gao, and F Fang. 2019. “First Report of Curvularia Intermedia Causing Leaf Blight on Annual Ryegrass (Lolium Multiflorum) in China.” *Plant Disease* 103(3): 585. https://doi.org/10.1094/PDIS-06-18-0955-PDN.

Liao, Y M, Z X Wang, M C Wei, and C Wang. 2020. “First Report of Phyllosticta Capitalensis Causing Black Spot Disease on Psidium Guajava in Mainland China.” *Plant Disease* 104(12): 3252. https://doi.org/10.1094/PDIS-02-20-0338-PDN.

Limtong, Savitree, Parichat Into, and Panchapond Attarat. 2020. “Biocontrol of Rice Seedling Rot Disease Caused by Curvularia Lunata and Helminthosporium Oryzae by Epiphytic Yeasts from Plant Leaves.” *Microorganisms* 8(5): 647. https://www.mdpi.com/2076-2607/8/5/647.

Liou, Guey Y, and Shean S Tzean. 1997. “Phylogeny of the Genus Arthrobotrys and Allied Nematode-Trapping Fungi Based on RDNA Sequences.” *Mycologia* 89(6): 876–84. https://doi.org/10.1080/00275514.1997.12026858.

Liu, L M et al. 2021. “Nigrospora Oryzae Causing Panicle Branch Rot Disease on Oryza Sativa (Rice).” *Plant Disease* 105(9): 2724. https://doi.org/10.1094/PDIS-11-20-2423-PDN.

Liu, Wei-Lun et al. 2019. “Clinical Manifestations of Candidemia Caused by Uncommon Candida Species and Antifungal Susceptibility of the Isolates in a Regional Hospital in Taiwan, 2007–2014.” *Journal of Microbiology, Immunology and Infection* 52(4): 612–19. https://www.sciencedirect.com/science/article/pii/S1684118217301883.

López-Fernández, Loida et al. 2018. “Understanding Mucor Circinelloides Pathogenesis by Comparative Genomics and Phenotypical Studies.” *Virulence* 9(1): 707–20.

Lu, W J, L H Wang, Y Q Wang, and C H Li. 2015. “First Report of Powdery Mildew Caused by Erysiphe Polygoni on Buckwheat in Yunnan, China.” *Plant Disease* 99(9): 1281. https://doi.org/10.1094/PDIS-12-14-1334-PDN.

Manamgoda, D. S. et al. 2014. “The Genus Bipolaris.” *Studies in Mycology* 79(1): 221–88. http://dx.doi.org/10.1016/j.simyco.2014.10.002.

Marin-Felix, Y., M. Hernández-Restrepo, and P. W. Crous. 2020. “Multi-Locus Phylogeny of the Genus Curvularia and Description of Ten New Species.” *Mycological Progress* 19(6): 559–88.

Matheny, P Brandon et al. 2018. “Revision of Pyrophilous Taxa of Pholiota Described from North America Reveals Four Species—P. Brunnescens, P. Castanea, P. Highlandensis, and P. Molesta.” *Mycologia* 110(6): 997–1016. https://doi.org/10.1080/00275514.2018.1516960.

McKinnon, Aimee C. et al. 2018. “Detection of the Entomopathogenic Fungus Beauveria Bassiana in the Rhizosphere of Wound-Stressed Zea Mays Plants.” *Frontiers in Microbiology* 9(JUN): 1–16.

McTaggart, A.R. et al. 2012. “A Review of the <I>Ustilago-Sporisorium-Macalpinomyces</I> Complex.” *Persoonia - Molecular Phylogeny and Evolution of Fungi* 29(1): 55–62. http://openurl.ingenta.com/content/xref?genre=article&issn=0031-5850&volume=29&issue=1&spage=55.

Méndez-Zamora, Andrés et al. 2020. “The Non-Saccharomyces Yeast Pichia Kluyveri for the Production of Aromatic Volatile Compounds in Alcoholic Fermentation.” *FEMS Yeast Research* 20(8): 1–14.

Mirić, Milenko, and Mimica Stefanović. 2018. “The Spread of Four Wood - Decaying Fungi through Artificially Infected Healthy Trees of Pedunculate Oak (Quercus Robur L.) in Vivo.” *Шумарство / Forestry / Sylviculture / Forstwesen* 70(1–2): 79–90. http://www.srpskosumarskoudruzenje.org.rs/pdf/sumarstvo/2018_1-2/sumarstvo2018_1-2_rad05.pdf.

Mnyone, Ladslaus L. et al. 2012. “Entomopathogenic Fungi, Metarhizium Anisopliae and Beauveria Bassiana Reduce the Survival of Xenopsylla Brasiliensis Larvae (Siphonaptera: Pulicidae).” *Parasites & Vectors* 5(1): 204. https://parasitesandvectors.biomedcentral.com/articles/10.1186/1756-3305-5-204.

Moiseenko, Konstantin et al. 2020. “Data on the Genome Analysis of the Wood-Rotting Fungus Steccherinum Ochraceum LE-BIN 3174.” *Data in Brief* 29: 105169. https://www.sciencedirect.com/science/article/pii/S2352340920300639.

Moiseenko, Konstantin V. et al. 2019. “Fungal Adaptation to the Advanced Stages of Wood Decomposition: Insights from the Steccherinum Ochraceum.” *Microorganisms* 7(11): 527. https://www.mdpi.com/2076-2607/7/11/527.

Montri, P., P. W. J. Taylor, and O. Mongkolporn. 2009. “Pathotypes of Colletotrichum Capsici , the Causal Agent of Chili Anthracnose, in Thailand.” *Plant Disease* 93(1): 17–20. https://apsjournals.apsnet.org/doi/10.1094/PDIS-93-1-0017.

Moslem, Mohmed, Kamel Abd-Elsalam, Mohamed Yassin, and Ali Bahkali. 2010. “First Morphomolecular Identification of Penicillium Griseofulvum and Penicillium Aurantiogriseum Toxicogenic Isolates Associated with Blue Mold on Apple.” *Foodborne Pathogens and Disease* 7(7): 857–61. https://doi.org/10.1089/fpd.2009.0507.

Naik, M K et al. 2017. “Characterization of Phytotoxin Producing Alternaria Species Isolatedfrom Sesame Leavesand Their Toxicity.” *Indian journal of experimental biology* 55(1): 36–43. http://www.ncbi.nlm.nih.gov/pubmed/30183227.

Novaković, Aleksandra et al. 2018. “Nutritional and Phenolic Profile of Small Edible Fungal Species Coprinellus Disseminatus (Pers.) J.E. Lange 1938.” *Food and Feed Research* 45(7): 119–28. https://scindeks.ceon.rs/Article.aspx?artid=2217-53691807119N.

NÚÑEZ, FÉLIX et al. 2000. “Effects of Substrate, Water Activity, and Temperature on Growth and Verrucosidin Production by Penicillium Polonicum Isolated from Dry-Cured Ham.” *Journal of Food Protection* 63(2): 231–36. https://meridian.allenpress.com/jfp/article/63/2/231/169219/Effects-of-Substrate-Water-Activity-and.

O’Brien, Caoimhe E. et al. 2018. “Genome Analysis of the Yeast Diutina Catenulata, a Member of the Debaryomycetaceae/Metschnikowiaceae (CTG-Ser) Clade.” *PLoS ONE* 13(6): 1–12.

Okolo, Ojogba Mark et al. 2015. “First Report of Neonatal Sepsis Due to Moesziomyces Bullatus in a Preterm Low‐birth‐weight Infant.” *JMM Case Reports* 2(2): 1–4. https://www.microbiologyresearch.org/content/journal/jmmcr/10.1099/jmmcr.0.000011.

Patil, Pravin D., and Ganapati D. Yadav. 2018. “Comparative Studies of White-Rot Fungal Strains ( Trametes Hirsuta MTCC-1171 and Phanerochaete Chrysosporium NCIM-1106) for Effective Degradation and Bioconversion of Ferulic Acid.” *ACS Omega* 3(11): 14858–68. https://pubs.acs.org/doi/10.1021/acsomega.8b01614.

Pedrini, Nicolás et al. 2013. “Targeting of Insect Epicuticular Lipids by the Entomopathogenic Fungus Beauveria Bassiana: Hydrocarbon Oxidation within the Context of a Host-Pathogen Interaction.” *Frontiers in Microbiology* 4: 1–18. http://journal.frontiersin.org/article/10.3389/fmicb.2013.00024/abstract.

Peksa, Kintija, and Biruta Bankina. 2019. “Characterization of Puccinia Recondita, the Causal Agent of Brown Rust: A Review.” In *Research for Rural Development*, , 70–76. https://llufb.llu.lv/conference/Research-for-Rural-Development/2019/LatviaResRuralDev_25th_2019_vol2-70-76.pdf.

Petrie, G A, and T C Vanterpool. 1978. “Mycosphaerella Tassiana on Cruciferae in Western Canada.” *Canadian Plant DiseaseSurvey* 58(4): 77–79.

Pinto, Cátia et al. 2018. “Understand the Potential Role of Aureobasidium Pullulans, a Resident Microorganism from Grapevine, to Prevent the Infection Caused by Diplodia Seriata.” *Frontiers in Microbiology* 9(DEC): 1–15.

Pitt, Wayne M., José Ramón Úrbez-Torres, and Florent P. Trouillas. 2015. “Dothiorella and Spencermartinsia, New Species and Records from Grapevines in Australia.” *Australasian Plant Pathology* 44(1): 43–56.

Poloni, Alana, and Jan Schirawski. 2016. “Host Specificity in Sporisorium Reilianum Is Determined by Distinct Mechanisms in Maize and Sorghum.” *Molecular Plant Pathology* 17(5): 741–54.

Pornsuriya, Chaninun, Thanunchanok Chairin, Narit Thaochan, and Anurag Sunpapao. 2017. “Choanephora Rot Caused by Choanephora Cucurbitarum on Brassica Chinensis in Thailand.” *Australasian Plant Disease Notes* 12(1): 13–15.

Pronczuk, M., J. Bojanowski, and R. Warzecha. 2004. “Effect of Leaf Infection by Kabatiella Zeae on Stalk Rot Prevalence and Grain Yield of Maize Hybrids.” *Journal of Phytopathology* 152(7): 410–15. https://onlinelibrary.wiley.com/doi/10.1111/j.1439-0434.2004.00864.x.

Quaedvlieg, W. et al. 2011. “<I>Zymoseptoria</I> Gen. Nov.: A New Genus to Accommodate <I>Septoria-</I>like Species Occurring on Graminicolous Hosts.” *Persoonia - Molecular Phylogeny and Evolution of Fungi* 26(1): 57–69. http://openurl.ingenta.com/content/xref?genre=article&issn=0031-5850&volume=26&issue=1&spage=57.

Ragavendran, Chinnasamy et al. 2019. “Larvicidal, Histopathological, Antibacterial Activity of Indigenous Fungus Penicillium Sp. Against Aedes Aegypti L and Culex Quinquefasciatus (Say) (Diptera: Culicidae) and Its Acetylcholinesterase Inhibition and Toxicity Assessment of Zebrafish (Danio Re.” *Frontiers in Microbiology* 10(MAR): 1–17.

Rao, Ravella Sreenivas, Bhaskar Bhadra, Neradugomma Naveen Kumar, and Sisinthy Shivaji. 2007. “Candida Hyderabadensis Sp. Nov., a Novel Ascomycetous Yeast Isolated from Wine Grapes.” *FEMS Yeast Research* 7(3): 489–93. https://doi.org/10.1111/j.1567-1364.2006.00206.x.

Rasgon, Jason L. 2011. “Using Infections to Fight Infections: Paratransgenic Fungi Can Block Malaria Transmission in Mosquitoes.” *Future microbiology* 6(8): 851–53.

Rinaldi, M G et al. 1987. “Human Curvularia Infections: Report of Five Cases and Review of the Literature.” *Diagnostic Microbiology and Infectious Disease* 6(1): 27–39. https://www.sciencedirect.com/science/article/pii/0732889387901118.

Rippon, J W, P M Arnow, R A Larson, and K L Zang. 1985. “‘Golden Tongue’ Syndrome Caused by Ramichloridium Schulzeri.” *Archives of dermatology* 121(7): 892–94. http://www.ncbi.nlm.nih.gov/pubmed/3860191.

Rivedal, Hannah M., Alexandra G. Stone, Paul M. Severns, and Kenneth B. Johnson. 2020. “Characterization of the Fungal Community Associated with Root, Crown, and Vascular Symptoms in an Undiagnosed Yield Decline of Winter Squash.” *Phytobiomes Journal* 4(2): 178–92. https://apsjournals.apsnet.org/doi/10.1094/PBIOMES-11-18-0056-R.

Rodriguez-Palacios, Alexander et al. 2016. “Clinical Effects of Gamma-Radiation-Resistant *Aspergillus Sydowii* on Germ-Free Mice Immunologically Prone to Inflammatory Bowel Disease” ed. Martin Hoenigl. *Journal of Pathogens* 2016: 5748745. https://doi.org/10.1155/2016/5748745.

Rolshausen, Philippe E. et al. 2014. “Identification of Eutypa Spp. Causing Eutypa Dieback of Grapevine in Eastern North America.” *Plant Disease* 98(4): 483–91. https://apsjournals.apsnet.org/doi/10.1094/PDIS-08-13-0883-RE.

Rooney-Latham, Suzanne et al. 2017. “Entyloma Helianthi: Identification and Characterization of the Causal Agent of Sunflower White Leaf Smut.” *Mycologia* 109(3): 520–28. https://doi.org/10.1080/00275514.2017.1362314.

Rudramurthy, Shivaprakash M. et al. 2019. “Invasive Aspergillosis by Aspergillus Flavus: Epidemiology, Diagnosis, Antifungal Resistance, and Management.” *Journal of Fungi* 5(55). https://www.mdpi.com/2309-608X/5/3/55.

Samson, R. A., and B. v.d. Lustgraaf. 1978. “Aspergillus Penicilloides and Eurotium Halophilicum in Association with House-Dust Mites.” *Mycopathologia* 64(1): 13–16.

Saroj, A et al. 2012. “First Report of Wet Rot of Withania Somnifera Caused by Choanephora Cucurbitarum in India.” *Plant Disease* 96(2): 293–293. https://doi.org/10.1094/PDIS-09-11-0801.

Satianpakiranakorn, Petlada, Pannida Khunnamwong, and Savitree Limtong. 2020. “Yeast Communities of Secondary Peat Swamp Forests in Thailand and Their Antagonistic Activities against Fungal Pathogens Cause of Plant and Postharvest Fruit Diseases.” *PLoS ONE* 15(3): 1–18. http://dx.doi.org/10.1371/journal.pone.0230269.

Saxena, Amrita, Richa Raghuwanshi, Vijai Kumar Gupta, and Harikesh B. Singh. 2016. “Chilli Anthracnose: The Epidemiology and Management.” *Frontiers in Microbiology* 7(SEP): 1–18.

SCHWARZE, F W M R, and S FINK. 1998. “Host and Cell Type Affect the Mode of Degradation by Meripilus Giganteus.” *New Phytologist* 139(4): 721–31. https://www.cambridge.org/core/article/host-and-cell-type-affect-the-mode-of-degradation-by-meripilus-giganteus/1989CB688EB39BB52F29DBF8C5E75733.

Singh, Saytandra, Rajesh Kumar Pandey, and B K Goswami. 2013. “Bio-Control Activity of Purpureocillium Lilacinum Strains in Managing Root-Knot Disease of Tomato Caused by Meloidogyne Incognita.” *Biocontrol Science and Technology* 23(12): 1469–89. https://doi.org/10.1080/09583157.2013.840770.

Slippers, B., T. A. Coutinho, B. D. Wingfield, and M. J. Wingfield. 2003. “A Review of the Genus Amylostereum and Its Association with Woodwasps.” *South African Journal of Science* 99(1–2): 70–74.

Smith, Wendy M., Gary Fahle, Robert B. Nussenblatt, and Hatice Nida Sen. 2013. “A Rare Case of Endogenous Aspergillus Conicus Endophthalmitis in an Immunocompromised Patient.” *Journal of Ophthalmic Inflammation and Infection* 3(1): 37. https://joii-journal.springeropen.com/articles/10.1186/1869-5760-3-37.

Soler-Hurtado, M. Mar, José Vladimir Sandoval-Sierra, Annie Machordom, and Javier Diéguez-Uribeondo. 2016. “Aspergillus Sydowii and Other Potential Fungal Pathogens in Gorgonian Octocorals of the Ecuadorian Pacific.” *PLoS ONE* 11(11): 1–12.

Somarathne, Magalla Bastian Chalitha Lakmal et al. 2018. “Functional Analysis of a Novel Parasitic Nematode-Specific Protein of Setaria Digitata Larvae in Culex Quinquefasciatus by SiRNA Mediated RNA Interference.” *Parasites & Vectors* 11(1): 541. https://parasitesandvectors.biomedcentral.com/articles/10.1186/s13071-018-3096-x.

SONG, YU et al. 2013. “Two New Species of Pestalotiopsis from Southern China.” *Phytotaxa* 126(1): 22. https://biotaxa.org/Phytotaxa/article/view/phytotaxa.126.1.2.

Spilker, D A, and P O Larsen. 1985. “Characterization and Host Range of Drechslera Catenaria, the Pathogen of Leaf Blight and Crown Rot of Creeping Bentgrass.” *Plant disease* 69(4): 331–33.

Spooner, B.M., and N.W. legon. 2006. “Additions and Amendments to the List of British Smut Fungi.” *Mycologist* 20(3): 90–96. https://linkinghub.elsevier.com/retrieve/pii/S0269915X06000322.

Stoll, Matthias, Dominik Begerow, and Franz Oberwinkler. 2005. “Molecular Phylogeny of Ustilago, Sporisorium, and Related Taxa Based on Combined Analyses of RDNA Sequences.” *Mycological Research* 109(3): 342–56. https://linkinghub.elsevier.com/retrieve/pii/S0953756208614227.

Su, G., S.-O. Suh, R. W. Schneider, and J. S. Russin. 2001. “Host Specialization in the Charcoal Rot Fungus, Macrophomina Phaseolina.” *Phytopathology®* 91(2): 120–26. https://apsjournals.apsnet.org/doi/10.1094/PHYTO.2001.91.2.120.

Suh, Sung-Oui, Nhu H Nguyen, and Meredith Blackwell. 2006. “A Yeast Clade near Candida Kruisii Uncovered: Nine Novel Candida Species Associated with Basidioma-Feeding Beetles.” *Mycological Research* 110(12): 1379–94. https://linkinghub.elsevier.com/retrieve/pii/S0953756206002711.

Thangaraj, K et al. 2018. “Report of Phoma Herbarum Causing Leaf Spot Disease of Camellia Sinensis in China.” *Plant Disease* 102(11): 2373–2373. https://doi.org/10.1094/PDIS-01-18-0121-PDN.

Trinh, Duy Nam, Thi Kim Lien Ha, and Dewen Qiu. 2020. “Biocontrol Potential of Some Entomopathogenic Fungal Strains Against Bean Aphid Megoura Japonica (Matsumura).” *Agriculture* 10(4): 114. https://www.mdpi.com/2077-0472/10/4/114.

Ullah, Najeeb, Khalid Pervaiz Akhtar, Muhammad Jawad Asghar, and Ghulam Abbas. 2019. “First Report of Macrophomina Phaseolina Causing Dry Root Rot of Lentil in Pakistan.” *Journal of Plant Pathology* 101(2): 429–429. https://doi.org/10.1007/s42161-018-00202-5.

Vabeikhokhei, Josiah M.C., Zoh Mangaiha, John Zothanzama, and H. Lalrinawmi. 2019. “Diversity Study of Wood Rotting Fungi from Two Different Forests in Mizoram, India.” *International Journal of Current Microbiology and Applied Sciences* 8(04): 2775–85. https://www.ijcmas.com/abstractview.php?ID=12766&vol=8-4-2019&SNo=323.

Vares, Tamara, Outi Niemenmaa, and Annele Hatakka. 1994. “Secretion of Ligninolytic Enzymes and Mineralization of 14 C-Ring-Labelled Synthetic Lignin by Three Phlebia Tremellosa Strains.” *Applied and Environmental Microbiology* 60(2): 569–75. https://journals.asm.org/doi/10.1128/aem.60.2.569-575.1994.

Vasina, Daria V., Konstantin V. Moiseenko, Tatiana V. Fedorova, and Tatiana V. Tyazhelova. 2017. “Lignin-Degrading Peroxidases in White-Rot Fungus Trametes Hirsuta 072. Absolute Expression Quantification of Full Multigene Family.” *PLoS ONE* 12(3): 1–16.

Vellanki, Sandeep et al. 2020. “A Novel Resistance Pathway for Calcineurin Inhibitors in the Human-Pathogenic Mucorales Mucor Circinelloides” ed. Michael Lorenz. *mBio* 11(1): e02949-19. https://journals.asm.org/doi/10.1128/mBio.02949-19.

Visalakshi, M. et al. 2020. “Studies on Mycosis of Metarhizium (Nomuraea) Rileyi on Spodoptera Frugiperda Infesting Maize in Andhra Pradesh, India.” *Egyptian Journal of Biological Pest Control* 30(1): 135. https://ejbpc.springeropen.com/articles/10.1186/s41938-020-00335-9.

Voegele, Ralf T. 2006. “Uromyces Fabae : Development, Metabolism, and Interactions with Its Host Vicia Faba.” *FEMS Microbiology Letters* 259(2): 165–73. https://academic.oup.com/femsle/article-lookup/doi/10.1111/j.1574-6968.2006.00248.x.

WANG, Ying et al. 2018. “Identification and Characterization of Pichia Membranifaciens Hmp-1 Isolated from Spoilage Blackberry Wine.” *Journal of Integrative Agriculture* 17(9): 2126–36. http://dx.doi.org/10.1016/S2095-3119(18)62027-1.

Welfringer, Anne et al. 2017. “A Rare Fungal Infection: Phaehyphomycosis Due to Veronaea Botryosa and Review of Literature.” *Medical Mycology Case Reports* 15(October 2016): 21–24. http://dx.doi.org/10.1016/j.mmcr.2017.02.001.

Whitby, Scott, Ernest C Madu, and Michael S Bronze. 1996. “Candida Zeylanoides Infective Endocarditis Complicating Infection With the Human Immunodeficiency Virus.” *The American Journal of the Medical Sciences* 312(3): 138–39. http://content.wkhealth.com/linkback/openurl?sid=WKPTLP:landingpage&an=00000441-199609000-00010.

WHO. 2018. “Mycotoxins, Fact Sheets.” https://www.who.int/news-room/fact-sheets/detail/mycotoxins (October 22, 2022).

Xie, Ming et al. 2015. “Persistence and Viability of Lecanicillium Lecanii in Chinese Agricultural Soil” ed. Gotthard Kunze. *PLOS ONE* 10(9): e0138337. https://dx.plos.org/10.1371/journal.pone.0138337.

Xu, J., X.-D. Xu, Y.-Y. Cao, and W.-M. Zhang. 2014. “First Report of Greenhouse Tomato Wilt Caused by Plectosphaerella Cucumerina in China.” *Plant Disease* 98(1): 158–158. https://doi.org/10.1094/PDIS-05-13-0566-PDN.

Yamin, Dina Hussein, Azlan Husin, and Azian Harun. 2021. “Risk Factors of Candida Parapsilosis Catheter-Related Bloodstream Infection.” *Frontiers in Public Health* 9(August): 1–11. https://www.frontiersin.org/articles/10.3389/fpubh.2021.631865/full.

Yang, Jinkui et al. 2011. “Genomic and Proteomic Analyses of the Fungus Arthrobotrys Oligospora Provide Insights into Nematode-Trap Formation” ed. Alex Andrianopoulos. *PLoS Pathogens* 7(9): e1002179. https://dx.plos.org/10.1371/journal.ppat.1002179.

Yeo, Sumin, Myung K. Kim, and Hyoung T. Choi. 2008. “Increased Expression of Laccase by the Addition of Phthalates in Phlebia Tremellosa.” *FEMS Microbiology Letters* 278(1): 72–77. https://academic.oup.com/femsle/article-lookup/doi/10.1111/j.1574-6968.2007.00971.x.

Zajc, Janja et al. 2020. “Characterization of Aureobasidium Pullulans Isolates Selected as Biocontrol Agents Against Fruit Decay Pathogens.” *Fungal Genom Biol* 10(1): 163.

Zhang, L X et al. 2012. “First Report of Nigrospora Oryzae Causing Leaf Spot of Cotton in China.” *Plant Disease* 96(9): 1379. https://doi.org/10.1094/PDIS-04-12-0349-PDN.

Zhu, G. S., Z. N. Yu, Y. Gui, and Z. Y. Liu. 2008. “A Novel Technique for Isolating Orchid Mycorrhizal Fungi.” *Fungal Diversity* 33: 123–37.
