# Supplementary material for: Size-resolved fungal bioaerosol diversity over an Indian agricultural field and their ecosystem-health implications
Source: Front Microbiol. 2025 Dec 3;16:1648820. doi: 10.3389/fmicb.2025.1648820 (PMC12708607; doi:10.3389/fmicb.2025.1648820)
Supplement: Supplementary file 2 [file Table_2.DOCX]

**Supplementary Table S2:** Brief description of properties of the fungal OTUs identified as beneficial/useful fungi

| **Fungal species** | **Saprophytic/environmental species** | **Biotechnological and industrial species** | **Medicinal species** | **Edible species** | **References** |
| --- | --- | --- | --- | --- | --- |
| *Amauroascus kuehnii* | Common saprophytic/environmental fungi- isolated from animal dungs, soil, and keratinous surfaces of live or deceased animals with keratinophilic activity | - | - | - | (Chlebicki and Spisak 2016) |
| *Agaricus gennadii* | - | - | - | Salt-loving edible mushroom | (J. Wu, Liao, and Lin 2020) |
| *Agaricus rotalis* | Rare environmental mushroom | - | - | - | (Kerrigan et al. 2005) |
| *Articulospora proliferata* | Aquatic hyphomycetes | - | - | - | (Jooste et al. 1990) |
| *Aspergillus fumigatus* | Omnipresent saprophyte- plays vital role in decaying matters and maintains balance in carbon-nitrogen cycle | - | - | - | (Dagenais and Keller 2009) |
| *Aspergillus penicillioides* | Xerophilic saprophyte- Common indoor fungi, present in dust etc. especially papers that too foxing papers | - | - | - | (Stevenson et al. 2017) |
| *Aspergillus subversicolor* | Saprophyte - Commonly found in damp indoor environments, soil, plant debris, marine environment, and food products; often reported in dust and in water-damaged building materials, such as wallboards, insulation, textiles, ceiling tiles, and manufactured wood |  |  |  | (Jurjevic, Peterson, and Horn 2012) |
| *Aspergillus sydowii* | saprophytic fungi found in soil and contaminate food. Also causes death of sea fan corals | - | - | - | (RYPIEN and ANDRAS 2008) |
| *Asterostroma cervicolor* | Common environmental fungi having widespread distribution | - | - | - | (Kirk et al. 2008) |
| *Auricularia nigricans* | - | - | - | Edible jelly fungus mainly found in trees and mountains also called as cloud ear fungus | (Nadir, Ali, and Salih 2020) |
| *Battarrea phalloides* | Saprophytic mushroom which is at current risk of extinction | - | - | - | (Gargano, Venturella, and Ferraro 2021) |
| *Beauveria bassiana* | - | Used as a biological insecticide to control a number of pests such as termites, thrips, whiteflies, aphids, different beetles, bedbugs and malaria transmitting mosquitoes | - | - | (McKinnon et al. 2018; Pedrini et al. 2013) |
| *Bullera variabilis* | Ballistoconidium-forming environmental yeast | - | - | - | (NAKASE and SUZUKI 1987) |
| *Byssochlamys spectabilis* | - | Industrial strain associated with the spoilage of canned and fermented food | - | - | (Samson et al. 2009) |
| *Candida ethanolica* | - | Industrial fodder yeast cultivated on synthetic ethanol | - | - | (Rybářová, Štros, and Kocková-Kratochvílová 1980; Xing et al. 2018) |
| *Cerinomyces canadensis* | Environmental fungi distributed in temperate regions | - | - | - | (Kirk et al. 2008) |
| *Chlorophyllum globosum* | Environmental mushroom found in tropical region | - | - | - | (Ge et al. 2018) |
| *Chlorophyllum hortense* | Big fleshy environmental fungus (mushroom) that occurs commonly in man-made habitats especially in compost-enriched garden soil, lawns, and grazing pastures | - | - | - | (Vizzini et al. 2014) |
| *Coprinellus aureogranulatus* | A mushroom found in all environments | - | - | - | (Huang and Bau 2018) |
| *Coprinellus heptemerus* | Rare ink-cap environmental mushroom | - | - | - | (Redhead et al. 2001) |
| *Coprinellus heterosetulosus* | Environmental mushroom | - | - | - | (Gierczyk et al. 2011) |
| *Coprinellus verrucispermus* | Common saprophytic mushroom of wood chips, leaf-litter, and herbivores dung | - | - | - | (Redhead et al. 2001) |
| *Coprinopsis acuminata* | Commonly known as humpback inkcap; grows on herbivore dung | - | - | - | (Gierczyk et al. 2011) |
| *Coprinopsis gonophylla* | Environmental mushroom | - | - | - | (Redhead et al. 2001) |
| *Coprinopsis laanii* | Environmental mushroom that commonly grows on trees | - | - | - | (Redhead et al. 2001) |
| *Coprinopsis macrocephala* | Environmental mushroom found in horse dung | - | - | - | (Redhead et al. 2001) |
| *Cunninghamella echinulata* | A soil saprotroph forming rhizoids especially in the soil rich in nitrogen, phosphorus, and potassium | - | - | - | (de Souza et al. 2018) |
| *Cystobasidium lysinophilum* | Environmental fungi | - | - | - | (Q. M. Wang et al. 2015) |
| *Devriesia fici* | Fungi associate with marine algae and is a marine fungus. The genus has been transferred to *Neodevriesia* | - | - | - | (M. M. Wang et al. 2017) |
| *Dichotomocladium sphaerosporum* | Environmental fungi found in dung | - | - | - | (Benny and Benjamin 1993) |
| *Diutina catenulata* | Ascomyceteous yeast isolated from environmental source that generally acts as food contaminant | - | - | - | (O’Brien et al. 2018) |
| *Entoloma infula* | Environmental mushroom | - | - | - | (Kirk et al. 2008) |
| *Exidia japonica* | Saprophytic mushroom that grows in freshly fallen dead wood and produces gelatinous biocorp | - | - | - | (Spirin, Malysheva, and Larsson 2018) |
| *Flammulina velutipes* | - | - | - | A special edible mushrooms which is also called as velvet shank | (Tang et al. 2016) |
| *Fusarium penzigii* | Environmental fungi observed in soil and dead plant substrata | - | - | - | (Schroers et al. 2009) |
| *Fuscoporia senex* | Environmental fungi capable of decaying wood | - | - | - | (Jang et al. 2012) |
| *Galerina laevis* | Environmental mushroom that are toxic | - | - | - | (Enjalbert et al. 2004) |
| *Ganoderma lucidum* | - | - | Used as herbal medicine and has a long history of use for promoting health and longevity | - | (Unlu et al. 2016) |
| *Ganoderma sichuanense* | - | - | Flat polyporous medicinal mushroom that has nutritional and therapeutic values and has been used in ancient Asian medicine | - | (Yao et al. 2020) |
| *Geastrum schmidelii* | Environmental dwarf earthstar mushrooms that grow in alkaline rich soil or calcareous soil | - | - | - | (Jeppson, Nilsson, and Larsson 2013) |
| *Geastrum triplex* | An inedible fungus found in the detritus and leaf litter of hardwood forests | - | - | - | (Kirk et al. 2008) |
| *Gloeophyllum carbonarium* | Rare environmental basidiomycota | - | - | - | (Yu, Dai, and Wang 2004) |
| *Gymnopilus underwoodii* | Environmental mushroom that grows on wood | - | - | - | (Guzmán-Dávalos et al. 2003) |
| *Hannaella kunmingensis* | Environmental yeast-like fungi | - | - | - | (Han et al. 2017) |
| *Hannaella oryzae* | Environmental yeast associated with plants and soil | - | - | - | (Q. Li et al. 2021) |
| *Hansfordia pulvinata* | - | Antifungal activity against the phytopathogenic fungi *Cladosporium fulvum* of tomato plant | - | - | (Iida et al. 2018) |
| *Hyphoderma mutatum* | Environmental basidiomycetes that grow on trees | - | - | - | (Telleria et al. 2012) |
| *Hyphodontia niemelaei* | Environmental basidiomycetes | - | - | - | (Wu 2001) |
| *Hypholoma fasciculare* | Saprotrophic poisonous mushroom also known as sulfur tuft or clustered woodlover - a common woodland mushroom | - | - | - | (Demirel and Uzun 2004) |
| *Inocybe curvipes* | Poisonous mushrooms that occur in urban and sub-urban habitats. Also, found in trees and local environments | - | - | - | (Buyck and Eyssartier 1999) |
| *Irpex lacteus* | Common crust fungi found in tropical region | - | - | - | (Novotný et al. 2000) |
| *Kluyveromyces lactis* | - | Yeast used for genetic studies and industrial applications. It has the ability to assimilate lactose and convert it to lactic acid | - | - | (Fukuhara 2006) |
| *Knufia marmoricola* | Environmental fungi isolated from limestone. It is an extremotolerant rock inhabiting fungus | - | - | - | (OWCZAREK-KOŚCIELNIAK and STERFLINGER 2018; Roberts and Evans 2011) |
| *Lacrymaria lacrymabunda* | Grows in woodlands, gardens, and park are commonly known as weeping widow mushroom | - | - | - | (Roberts and Evans 2011) |
| *Lentinus squarrosulus* | - | - | - | Common edible mushroom with potent antioxidants | (Mhd Omar et al. 2011) |
| *Lenzites betulina* | - | - | Commonly known as gilled polypore, birch mazegill, or multicolor gill polypore. It has several medicinal properties, including antioxidant, antimicrobial, antitumor, and immunosuppressive activities. Mostly found on barks | - | (Liu et al. 2014) |
| *Leptodiscella africana* | Environmental fungi that grow in soil | - | - | - | (Madrid et al. 2012) |
| *Leucocoprinus birnbaumii* | Gilled mushroom commonly found in flower pots and plant pots | - | - | - | (Adikaram, Yakandawala, and Jayasinghe 2020) |
| *Metarhizium rileyi* | - | It is an entomopathogenic fungi used as biopesticide | - | - | (Binneck, Lastra, and Sosa-Gómez 2019) |
| *Morchella septimelata* | Environmental fungi | - | - | - | (Kuo et al. 2012) |
| *Mortierella exigua* | - | Saprophytic fungi found in soil which has the ability to undergo diverse bio-transformations or accumulation of unsaturated fatty acids making them attractive for biotechnological applications | - | - | (Vadivelan and Venkateswaran 2014) |
| *Myceliophthora thermophila* | - | A thermophilic fungus that grows at 45-50 C, efficiently degrades cellulose, and used in biofuel production | - | - | (J. Li et al. 2020) |
| *Mycothermus thermophilus* | - | Thermophilic fungi have received substantial attention in industry for their potential to produce thermostable enzymes and as production platforms tolerant of high temperatures | - | - | (Natvig et al. 2015) |
| *Myrmecridium schulzeri* | Uncommon soil saprophyte of worldwide distribution. It has also been isolated from plant detritus | - | - | - | (Rezakhani et al. 2019) |
| *Panaeolus antillarum* | Commonly seen wild grey mushroom that grows in dung | - | - | - | (Desjardin 2017) |
| *Panaeolus papilionaceus* | Common little brown mushroom that feeds on dung | - | - | - | (Murrill 1909) |
| *Papiliotrema terrestris* | - | Basidiomycota that produces β-galactosidase oligosaccharides | - | - | (Ke, Fulmer, and Mizutani 2018) |
| *Penicillium aurantiogriseum* | - | Biotechnologically important-cheese production | - | - | (Kandasamy et al. 2020) |
| *Penicillium citrinum* | - | - | Medicinal fungi | - | (Sharma et al. 2021) |
| *Penicillium dravuni* | A marine derived species especially from marine algae | - | - | - | (Janso et al. 2005) |
| *Penicillium multicolor* | - | - | Medicinal fungi produce antimycobacterial compound | - | (Hemtasin et al. 2016) |
| *Penicillium polonicum* | - | Produces penicillic acid, verucosidin, patulin, anacine, 3-methoxyviridicatin and glycopeptide | - | - | (Valente et al. 2021) |
| *Peniophorella pubera* | Environmental fungi | - | - | - | (Yurchenko, Wu, and Maekawa 2020) |
| *Peziza buxea* | An environmental cup-fungi appears in different color | - | - | - | (Kirk et al. 2008) |
| *Peziza vesiculosa* | It is found on nutrient-rich soils, rotting straw and manure and can often be seen on compost heaps. This species is considered poisonous | - | - | - | (Kirk et al. 2008) |
| *Phanerochaete chrysosporium* | Known as crust fungi and white rot fungi that degrades lignin | - | - | - | (Ganesh Kumar, Sekaran, and Krishnamoorthy 2006) |
| *Physcia dubia* | It is known as blue-gray rosette lichen and powder-back lichen. It is calcareous, basaltic, and siliceous. Grows on rocks, bones, barks, and soil. Very common in Europe, North America and New Zealand, and more patchily distributed in South America, Asia, Australia and Antarctica | - | - | - | (Sonina et al. 2017) |
| *Pichia kluyveri* | - | Yeast helps in fermentation of wine and improves wine quality | - | - | (Méndez-Zamora et al. 2020) |
| *Pichia membranifaciens* | - | Used in fermentation, an industrial strain that controls the growth of *Botrytis cinerea* that causes grey mold disease in grapevine | - | - | (Masih 2001) |
| *Pluteus petasatus* | - | - | - | Edible mushroom | (Justo et al. 2011) |
| *Psathyrella candolleana* | Commonly found in lawns | - | - | - | (Al-Habib, Holliday, and Tura 2014) |
| *Psathyrella phegophila* | Environmental basidiomycetes | - | - | - | (Voto, Dovana, and Garbelotto 2019) |
| *Psathyrella umbrina* | Environmental mushroom | - | - | - | (Frank, Coffan, and Southworth 2010) |
| *Pseudozyma hubeiensis* | - | Produces value added products like endoxylanase and β-xylosidase | - | - | (Mhetras, Liddell, and Gokhale 2016; Tanimura et al. 2016) |
| *Punctularia strigosozonata* | Environmental basidiomycetes otherwise called as tree bacons. White-rot fungi with powerful lignin degradation efficiency and wood decaying capabilities | - | - | - | (Kirk et al. 2008) |
| *Purpureocillium lilacinum* | Environmental fungi. It has been isolated from cultivated and uncultivated soils, forests, grassland, deserts, estuarine sediments and sewage sludge, and insects | - | - | - | (Chen, Lin, and Hung 2019) |
| *Pycnoporus cinnabarinus* | Rare polyporous Basidiomycota that occurs in cooler temperate regions especially on trees or woods | - | - | - | (Levasseur et al. 2014) |
| *Rasamsonia composticola* | Thermophilic species isolated from compost | - | - | - | (Su and Cai 2013) |
| *Rhodonia placenta* | Brown rot fungi, occurring in coniferous forest, and a potential decaying fungus | - | - | - | (Kölle et al. 2020) |
| *Ruinenia clavata* | Yeast or yeast-like *Pucciniomycotina* fungi | - | - | - | (Q.-M. Wang et al. 2015) |
| *Saccharomycopsis crataegensis* | Environmental heterothallic yeast | - | - | - | (Kurtzman and Wickerham 1973) |
| *Sakaguchia oryzae* | Environmental *Pucciniomycotina* fungi | - | - | - | (Q.-M. Wang et al. 2015) |
| *Schizophyllum commune* | - | - | Omnipresent medicinal mushroom. Especially seen in decaying trees after rain | - | (Arun, Eyini, and Gunasekaran 2015) |
| *Spiromastix princeps* | Xerotollerant fungi found in house dust | - | - | - | (Hirooka et al. 2016) |
| *Spizellomyces dolichospermus* | Found in soil and mainly in aquatic habitats | - | - | - | (Wakefield et al. 2010) |
| *Sporobolomyces bannaensis* | Environmental ballistoconidium forming yeast | - | - | - | (Zhao 2003) |
| *Sporobolomyces phaffii* | Environmental basidiomycetes | - | - | - | (WANG and BAI 2004) |
| *Stereum hirsutum* | Also called false turkey tail and hairy curtain crust. It is a fungus typically forming multiple brackets on dead wood | - | - | - | (Grass et al. 2011) |
| *Talaromyces euchlorocarpius* | Soil fungi | - | - | - | (Yilmaz et al. 2014) |
| *Talaromyces sayulitensis* | Grows in mineral rich substrates like oil shale | - | - | - | (de Goes et al. 2017; Jiang et al. 2018) |
| *Thermoascus aurantiacus* | - | Secrete enzymes that deconstruct biomass at high temperatures | - | - | (McClendon et al. 2012) |
| *Thermomyces dupontii* | - | Produces low molecular weight thermo-alkali-stable and mercury ion-tolerant xylanase | - | - | (Seemakram et al. 2020) |
| *Tilletiopsis washingtonensis* | Saprophytic yeast-like fungi | - | - | - | (Richter et al. 2019) |
| *Tomentellopsis bresadolana* | Environmental fungi that grow on wood | - | - | - | (Ordynets et al. 2017) |
| *Trametes versicolor* | - | - | Medicinal basidiomycetes | - | (Knežević et al. 2018) |
| *Trichoderma reesei* | Mesophilic filamentous fungi, secretes large quantities of cellulolytic enzymes like cellulase and hemicellulase | - | - | - | (Fonseca, Parreiras, and Murakami 2020; Rantasalo et al. 2019) |
| *Trichothecium crotocinigenum* | - | - | Medicinal value – produces antimicrobial compounds | - | (Yang et al. 2018) |
| *Virgaria nigra* | - | Biotechnologically important strain- produces 2,7-dihydroxy naphthalene, virgaricin B, and virgaricin | - | - | (ANDO, YOSHIDA, and OKUHARA 1988; Samy et al. 2022) |

**References**

Adikaram, N.K.B., D.M.D. Yakandawala, and L. Jayasinghe. 2020. “*Leucocoprinus Birnbaumii* (Agaricales: Basidiomycota), Attractive Yellow Houseplant Mushroom, Revisited after 100 Years.” *Ceylon Journal of Science* 49(2): 209.

Al-Habib, Mouthana N, John C Holliday, and Daniel Tura. 2014. “The Pale Brittle Stem Mushroom, Psathyrella Candolleana (Higher Basidiomycetes): An Indigenous Medicinal Mushroom New to Iraq.” *International Journal of Medicinal Mushrooms* 16(6): 617–22. http://www.dl.begellhouse.com/journals/708ae68d64b17c52,077f1bbb3fcde585,6ea97f716c685cf0.html.

Ando, Takeshi, Keizo Yoshida, And Masakuni Okuhara. 1988. “Vinigrol, A Novel Antihypertensive And Platelet Aggregation Inhibitory Agent Produced By A Fungus, Virgaria Nigra Ii. Pharmacological Characteristics.” *The Journal of Antibiotics* 41(1): 31–35.

Arun, G, M Eyini, and P Gunasekaran. 2015. “Characterization and Biological Activities of Extracellular Melanin Produced by Schizophyllum Commune (Fries).”

Benny, Gerald L, and R K Benjamin. 1993. “Observations on Thamnidiaceae (Mucorales). VI. Two New Species of Dichotomocladium and the Zygospores of D. Hesseltinei (Chaetocladiaceae).” *Mycologia* 85(4): 660–71.

Binneck, Eliseu, Claudia Cristina López Lastra, and Daniel R. Sosa-Gómez. 2019. “ Genome Sequence of Metarhizium Rileyi , a Microbial Control Agent for Lepidoptera .” *Microbiology Resource Announcements* 8(36): 14–16.

Buyck, Bart, and Guillaume Eyssartier. 1999. “Two New Species of Inocybe (Cortinariaceae) from African Woodland.” *Kew Bulletin*: 675–81.

Chen, W, S Lin, and S Hung. 2019. “Successful Treatment of Recurrent Cutaneous Purpureocillium Lilacinum (Paecilomyces Lilacinus) Infection with Posaconazole and Surgical Debridement.” *Acta Dermato Venereologica* 99(13): 1313–14. https://medicaljournalssweden.se/actadv/article/view/3455.

Chlebicki, Andrzej, and Wojciech Spisak. 2016. “Amauroascus Kuehnii and Other Fungi Isolated from a Deer Horn in Poland.” *Polish Botanical Journal* 61(1): 161–66.

Dagenais, Taylor R. T., and Nancy P. Keller. 2009. “Pathogenesis of Aspergillus Fumigatus in Invasive Aspergillosis.” *Clinical Microbiology Reviews* 22(3): 447–65. https://doi.org/10.1128/CMR.00055-08.

Demirel, Kenan, and Yusuf Uzun. 2004. “Some Poisonous Fungi of East Anatolia.” *Turkish Journal of Botany* 28(1–2): 215–19.

Desjardin, DE. 2017. “Panaeolus Antillarum (Basidiomycota, Psathyrellaceae) from Wild Elephant Dung in Thailand.” *Current Research in Environmental & Applied Mycology* 7(4): 275–81. http://www.creamjournal.org/pdf/CREAM_7_4_4.pdf.

Enjalbert, Françoise et al. 2004. “Amatoxins in Wood-Rotting Galerina Marginata.” *Mycologia* 96(4): 720–29. https://www.tandfonline.com/doi/full/10.1080/15572536.2005.11832920.

Fonseca, Lucas Miranda, Lucas Salera Parreiras, and Mario Tyago Murakami. 2020. “Rational Engineering of the Trichoderma Reesei RUT-C30 Strain into an Industrially Relevant Platform for Cellulase Production.” *Biotechnology for Biofuels* 13(1): 1–15. https://doi.org/10.1186/s13068-020-01732-w.

Frank, Jonathan L, Robert A Coffan, and Darlene Southworth. 2010. “Aquatic Gilled Mushrooms: Psathyrella Fruiting in the Rogue River in Southern Oregon.” *Mycologia* 102(1): 93–107. https://doi.org/10.3852/07-190.

Fukuhara, Hiroshi. 2006. “Kluyveromyces Lactis Â€“ a Retrospective.” *FEMS Yeast Research* 6(3): 323–24. https://academic.oup.com/femsyr/article-lookup/doi/10.1111/j.1567-1364.2005.00012.x.

Ganesh Kumar, A., G. Sekaran, and Sarayu Krishnamoorthy. 2006. “Solid State Fermentation of Achras Zapota Lignocellulose by Phanerochaete Chrysosporium.” *Bioresource Technology* 97(13): 1521–28. https://linkinghub.elsevier.com/retrieve/pii/S0960852405003226.

Gargano, Maria Letizia, Giuseppe Venturella, and Valeria Ferraro. 2021. “Is Battarrea Phalloides Really an Endangered Species?” *Plant Biosystems - An International Journal Dealing with all Aspects of Plant Biology* 155(4): 759–62. https://doi.org/10.1080/11263504.2020.1779847.

Ge, Zai Wei et al. 2018. “A Multi-Gene Phylogeny of Chlorophyllum (Agaricaceae, Basidiomycota): New Species, New Combination and Infrageneric Classification.” *MycoKeys* 32: 65–90.

Gierczyk, Błażej et al. 2011. “Rare Species of the Genus Coprinus Pers. s. Lato.” *Acta Mycologica* 46(1).

de Goes, Kelly C. G. P. et al. 2017. “Talaromyces Sayulitensis, Acidiella Bohemica and Penicillium Citrinum in Brazilian Oil Shale by-Products.” *Antonie van Leeuwenhoek* 110(12): 1637–46. http://link.springer.com/10.1007/s10482-017-0913-8.

Grass, Josephine et al. 2011. “Discovery and Structural Characterization of Fucosylated Oligomannosidic N-Glycans in Mushrooms.” *Journal of Biological Chemistry* 286(8): 5977–84. https://linkinghub.elsevier.com/retrieve/pii/S0021925820519716.

Guzmán-Dávalos, Laura et al. 2003. “Traditional Infrageneric Classification of Gymnopilus Is Not Supported by Ribosomal DNA Sequence Data.” *Mycologia* 95(6): 1204–14. https://www.jstor.org/stable/3761920?origin=crossref.

Han, Long et al. 2017. “Hannaella Dianchiensis Sp. Nov., a Basidiomycetous Yeast Species Isolated from Lake Water.” *International Journal of Systematic and Evolutionary Microbiology* 67(6): 2014–18. https://www.microbiologyresearch.org/content/journal/ijsem/10.1099/ijsem.0.001908.

Hemtasin, Chulida et al. 2016. “Bioactive Azaphilones from the Fungus Penicillium Multicolor CM01.” *Phytochemistry Letters* 16: 56–60. https://www.sciencedirect.com/science/article/pii/S1874390016300301.

Hirooka, Yuuri, Joey B. Tanney, Hai D.T. Nguyen, and Keith A. Seifert. 2016. “Xerotolerant Fungi in House Dust: Taxonomy of Spiromastix, Pseudospiromastix and Sigleria Gen. Nov. in Spiromastigaceae (Onygenales, Eurotiomycetes).” *Mycologia* 108(1): 135–56.

Huang, Mei, and Tolgor Bau. 2018. “New Findings of Coprinellus Species (Psathyrellaceae, Agaricales) in China.” *Phytotaxa* 374(2): 119–28.

Iida, Y. et al. 2018. “Evaluation of the Potential Biocontrol Activity of Dicyma Pulvinata against Cladosporium Fulvum , the Causal Agent of Tomato Leaf Mould.” *Plant Pathology* 67(9): 1883–90. https://onlinelibrary.wiley.com/doi/10.1111/ppa.12916.

Jang, Yeongseon et al. 2012. “Four Unrecorded Wood Decay Fungi from Seoul in Korea.” *Mycobiology* 40(3): 195–201. https://doi.org/10.5941/MYCO.2012.40.3.195.

Janso, Jeffrey E et al. 2005. “Penicillium Dravuni, a New Marine-Derived Species from an Alga in Fiji.” *Mycologia* 97(2): 444–53. https://doi.org/10.1080/15572536.2006.11832820.

Jeppson, Mikael, R. Henrik Nilsson, and Ellen Larsson. 2013. “European Earthstars in Geastraceae (Geastrales, Phallomycetidae)-a Systematic Approach Using Morphology and Molecular Sequence Data.” *Systematics and Biodiversity* 11(4): 437–65. http://dx.doi.org/10.1080/14772000.2013.857367.

Jiang, Xian Zhi, Zhong Dong Yu, Yong Ming Ruan, and Long Wang. 2018. “Three New Species of Talaromyces Sect. Talaromyces Discovered from Soil in China.” *Scientific Reports* 8(1): 1–11. http://dx.doi.org/10.1038/s41598-018-23370-x.

Jooste, W J, A Roldan, W J J Van Der Merwe, and M Honrubia. 1990. “Articulospora Proliferata Sp. Nov., an Aquatic Hyphomycete from South Africa and Spain.” *Mycological Research* 94(7): 947–51. https://www.sciencedirect.com/science/article/pii/S0953756209813105.

Jurjevic, Zeljko, Stephen W. Peterson, and Bruce W. Horn. 2012. “Aspergillus Section Versicolores: Nine New Species and Multilocus DNA Sequence Based Phylogeny.” *IMA Fungus* 3(1): 59–79.

Justo, Alfredo et al. 2011. “Species Recognition in Pluteus and Volvopluteus (Pluteaceae, Agaricales): Morphology, Geography and Phylogeny.” *Mycological Progress* 10(4): 453–79. http://link.springer.com/10.1007/s11557-010-0716-z.

Kandasamy, Sujatha et al. 2020. “Characterisation of Fungal Contamination Sources for Use in Quality Management of Cheese Production Farms in Korea.” *Asian-Australasian Journal of Animal Sciences* 33(6): 1002–11.

Ke, Qingdong, Preston Fulmer, and Atsushi Mizutani. 2018. “Toxicological Evaluation of β-Galactosidase Enzyme Produced by Papiliotrema Terrestris.” *Regulatory Toxicology and Pharmacology* 92: 213–19. https://linkinghub.elsevier.com/retrieve/pii/S0273230017303896.

Kerrigan, Richard W et al. 2005. “Agaricus Section Xanthodermatei: A Phylogenetic Reconstruction with Commentary on Taxa.” *Mycologia* 97(6): 1292–1315. https://doi.org/10.1080/15572536.2006.11832737.

Kirk, Paul M, Paul F Cannon, D W Minter, and J A Stalpers. 2008. “Dictionary of the Fungi.(10thedn).” *Wallingford, UK*.

Knežević, Aleksandar et al. 2018. “Antioxidative, Antifungal, Cytotoxic and Antineurodegenerative Activity of Selected Trametes Species from Serbia.” *PLoS ONE* 13(8): 1–18.

Kölle, Martina et al. 2020. “Degradative Capacity of Two Strains of Rhodonia Placenta: From Phenotype to Genotype.” *Frontiers in Microbiology* 11(June): 1–15.

Kuo, Michael et al. 2012. “Taxonomic Revision of True Morels (Morchella) in Canada and the United States.” *Mycologia* 104(5): 1159–77.

Kurtzman, C. P., and L. J. Wickerham. 1973. “Saccharomycopsis Crataegensis,.” *Antonie van Leeuwenhoek* 39: 81–87.

Levasseur, Anthony et al. 2014. “The Genome of the White-Rot Fungus Pycnoporus Cinnabarinus: A Basidiomycete Model with a Versatile Arsenal for Lignocellulosic Biomass Breakdown.” *BMC Genomics* 15(1): 486. http://bmcgenomics.biomedcentral.com/articles/10.1186/1471-2164-15-486.

Li, Jinyang et al. 2020. “Metabolic Engineering of the Cellulolytic Thermophilic Fungus Myceliophthora Thermophila to Produce Ethanol from Cellobiose.” *Biotechnology for Biofuels* 13(1): 1–15. https://doi.org/10.1186/s13068-020-1661-y.

Li, Qiang et al. 2021. “Characterization of the Complete Mitochondrial Genome of Basidiomycete Yeast Hannaella Oryzae: Intron Evolution, Gene Rearrangement, and Its Phylogeny.” *Frontiers in Microbiology* 12(May): 1–11.

Liu, Kun, Jun-Li Wang, Le Zhao, and Qian Wang. 2014. “Anticancer and Antimicrobial Activities and Chemical Composition of the Birch Mazegill Mushroom Lenzites Betulina (Higher Basidiomycetes).” *International Journal of Medicinal Mushrooms* 16(4): 327–37. http://www.dl.begellhouse.com/journals/708ae68d64b17c52,4c99b516238143af,5ba3b3f80fa91418.html.

Madrid, Hugo, Josepa Gené, Josep Cano, and Josep Guarro. 2012. “A New Species of Leptodiscella from Spanish Soil.” *Mycological Progress* 11(2): 535–41.

Masih, E. 2001. “Characterisation of the Yeast Pichia Membranifaciens and Its Possible Use in the Biological Control of Botrytis Cinerea, Causing the Grey Mould Disease of Grapevine.” *FEMS Microbiology Letters* 202(2): 227–32. http://doi.wiley.com/10.1016/S0378-1097(01)00323-8.

McClendon, Shara D. et al. 2012. “Thermoascus Aurantiacus Is a Promising Source of Enzymes for Biomass Deconstruction under Thermophilic Conditions.” *Biotechnology for Biofuels* 5(1): 54. https://biotechnologyforbiofuels.biomedcentral.com/articles/10.1186/1754-6834-5-54.

McKinnon, Aimee C. et al. 2018. “Detection of the Entomopathogenic Fungus Beauveria Bassiana in the Rhizosphere of Wound-Stressed Zea Mays Plants.” *Frontiers in Microbiology* 9(JUN): 1–16.

Méndez-Zamora, Andrés et al. 2020. “The Non-Saccharomyces Yeast Pichia Kluyveri for the Production of Aromatic Volatile Compounds in Alcoholic Fermentation.” *FEMS Yeast Research* 20(8): 1–14.

Mhd Omar, Nor Adila et al. 2011. “Nutritional Composition, Antioxidant Activities, and Antiulcer Potential of Lentinus Squarrosulus (Mont.) Mycelia Extract.” *Evidence-Based Complementary and Alternative Medicine* 2011.

Mhetras, Nutan, Susan Liddell, and Digambar Gokhale. 2016. “Purification and Characterization of an Extracellular β-Xylosidase from Pseudozyma Hubeiensis NCIM 3574 (PhXyl), an Unexplored Yeast.” *AMB Express* 6(1).

Murrill, William A. 1909. “A New Poisonous Mushroom.” *Mycologia* 1(5): 211. https://www.jstor.org/stable/3753515?origin=crossref.

Nadir, Hawrez A, Ari J Ali, and Salah A Salih. 2020. “Auricularia Nigricans (Auriculariaceae, Basidiomycota) Is First Introduced from Halabja Province, Iraq.” *The Journal of Fungus Nisan* 11(1): 68–74.

NAKASE, TAKASHI, and MOTOFUMI SUZUKI. 1987. “STUDIES ON BALLISTOSPORE-FORMING YEASTS FROM THE DEAD LEAVES OF MISCANTHUS SINENSIS WITH DESCRIPTIONS OF THE NEW SPECIES SPOROBOLOM YCES MISCANTHI, SPOROBOLOMYCES SUBROSEUS, AND SPOROBOLOMYCES WEIJMANII.” *The Journal of General and Applied Microbiology* 33(2): 177–96.

Natvig, Donald O et al. 2015. “Mycothermus Thermophilus Gen. et Comb. Nov., a New Home for the Itinerant Thermophile Scytalidium Thermophilum (Torula Thermophila).” *Mycologia* 107(2): 319–27. https://doi.org/10.3852/13-399.

Novotný, Č et al. 2000. “Irpex Lacteus , a White Rot Fungus Applicable to Water and Soil Bioremediation.” *Applied Microbiology and Biotechnology* 54(6): 850–53. http://link.springer.com/10.1007/s002530000432.

O’Brien, Caoimhe E. et al. 2018. “Genome Analysis of the Yeast Diutina Catenulata, a Member of the Debaryomycetaceae/Metschnikowiaceae (CTG-Ser) Clade.” *PLoS ONE* 13(6): 1–12.

Ordynets, Alexander et al. 2017. “Aphyllophoroid Fungi in Insular Woodlands of Eastern Ukraine.” *Biodiversity Data Journal* 5.

OWCZAREK-KOŚCIELNIAK, MAGDALENA, and KATJA STERFLINGER. 2018. “First Records of Knufia Marmoricola from Limestone Outcrops in the Wyżyna Krakowsko-Częstochowska Upland, Poland.” *Phytotaxa* 357(2): 94. https://biotaxa.org/Phytotaxa/article/view/phytotaxa.357.2.2.

Pedrini, Nicolás et al. 2013. “Targeting of Insect Epicuticular Lipids by the Entomopathogenic Fungus Beauveria Bassiana: Hydrocarbon Oxidation within the Context of a Host-Pathogen Interaction.” *Frontiers in Microbiology* 4: 1–18. http://journal.frontiersin.org/article/10.3389/fmicb.2013.00024/abstract.

Rantasalo, Anssi et al. 2019. “Novel Genetic Tools That Enable Highly Pure Protein Production in Trichoderma Reesei.” *Scientific Reports* 9(1): 1–12.

Redhead, Scott A et al. 2001. “Coprinus Pers. and the Disposition of Coprinus Species Sensu Lato.” *TAXON* 50(1): 203–41. https://onlinelibrary.wiley.com/doi/abs/10.2307/1224525.

Rezakhani, Forough et al. 2019. “A Preliminary Report of Aquatic Hyphomycetes Isolated from Anzali Lagoon (Gilan Province, North of Iran).” *Rostaniha* 20(2): 123–43.

Richter, Christian, Andrey M. Yurkov, Teun Boekhout, and Marc Stadler. 2019. “Diversity of Tilletiopsis-Like Fungi in Exobasidiomycetes (Ustilaginomycotina) and Description of Six Novel Species.” *Frontiers in Microbiology* 10(November): 1–14.

Roberts, Peter, and Shelley Evans. 2011. *The Book of Fungi*. Chicago, Illinois: University of Chicago Press.

Rybářová, Johanna, F Štros, and Anna Kocková-Kratochvílová. 1980. “Candida Ethanolica n. Sp.” *Zeitschrift für allgemeine Mikrobiologie* 20(9): 579–81. https://doi.org/10.1002/jobm.19800200906.

RYPIEN, KRYSTAL L, and JASON P ANDRAS. 2008. “Isolation and Characterization of Microsatellite Loci in Aspergillus Sydowii, a Pathogen of Caribbean Sea Fan Corals.” *Molecular Ecology Resources* 8(1): 230–32. https://onlinelibrary.wiley.com/doi/abs/10.1111/j.1471-8286.2007.01934.x.

Samson, R.A., J. Houbraken, J. Varga, and J.C. Frisvad. 2009. “Polyphasic Taxonomy of the Heat Resistant Ascomycete Genus <I>Byssochlamys</I> and Its <I>Paecilomyces</I> Anamorphs.” *Persoonia - Molecular Phylogeny and Evolution of Fungi* 22(1): 14–27. http://openurl.ingenta.com/content/xref?genre=article&issn=0031-5850&volume=22&issue=1&spage=14.

Samy, Mamdouh Nabil et al. 2022. “Elastase Inhibitory Activity of Secondary Metabolites from the Fungus Virgaria Nigra CF-231658.” *Natural Product Research* 36(6): 1668–71. https://www.tandfonline.com/doi/full/10.1080/14786419.2021.1899175.

Schroers, Hans-Josef et al. 2009. “Taxonomy and Phylogeny of the Fusarium Dimerum Species Group.” *Mycologia* 101(1): 44–70. https://doi.org/10.3852/08-002.

Seemakram, Wasan et al. 2020. “Purification, Characterization and Partial Amino Acid Sequences of Thermo-Alkali-Stable and Mercury Ion-Tolerant Xylanase from Thermomyces Dupontii KKU–CLD–E2–3.” *Scientific Reports* 10(1): 1–10. https://doi.org/10.1038/s41598-020-78670-y.

Sharma, Hemant, Arun Kumar Rai, Rajen Chettri, and Poonam Singh Nigam. 2021. “Bioactivites of Penicillium Citrinum Isolated from a Medicinal Plant Swertia Chirayita.” *Archives of Microbiology* 203(8): 5173–82. https://doi.org/10.1007/s00203-021-02498-x.

Sonina, Anzhella V., Anastasya D. Rumjantseva, Anna A. Tsunskaya, and Vera I. Androsova. 2017. “Adaptations of Epilithic Lichens to the Microclimate Conditions of the White Sea Coast.” *Czech Polar Reports* 7(2): 133–43. https://journals.muni.cz/CPR/article/view/12998.

de Souza, Patrícia et al. 2018. “Production of a Biosurfactant by Cunninghamella Echinulata Using Renewable Substrates and Its Applications in Enhanced Oil Spill Recovery.” *Colloids and Interfaces* 2(4): 63. http://www.mdpi.com/2504-5377/2/4/63.

Spirin, Viacheslav, Vera Malysheva, and Karl-Henrik Larsson. 2018. “On Some Forgotten Species of Exidia and Myxarium (Auriculariales, Basidiomycota).” *Nordic Journal of Botany* 36(3): njb-01601. https://onlinelibrary.wiley.com/doi/10.1111/njb.01601.

Stevenson, Andrew et al. 2017. “Aspergillus Penicillioides Differentiation and Cell Division at 0.585 Water Activity.” *Environmental Microbiology* 19(2): 687–97. https://onlinelibrary.wiley.com/doi/10.1111/1462-2920.13597.

Su, Yuan-Ying, and Lei Cai. 2013. “Rasamsonia Composticola, a New Thermophilic Species Isolated from Compost in Yunnan, China.” *Mycological Progress* 12(2): 213–21. http://link.springer.com/10.1007/s11557-012-0827-9.

Tang, Calyn et al. 2016. “Golden Needle Mushroom: A Culinary Medicine with Evidenced-Based Biological Activities and Health Promoting Properties.” *Frontiers in Pharmacology* 7(DEC).

Tanimura, Ayumi et al. 2016. “Lipid Production through Simultaneous Utilization of Glucose, Xylose, and l-Arabinose by Pseudozyma Hubeiensis: A Comparative Screening Study.” *AMB Express* 6(1): 58. https://amb-express.springeropen.com/articles/10.1186/s13568-016-0236-6.

Telleria, M. Teresa et al. 2012. “A New Species of Hyphoderma (Meruliaceae, Polyporales) and Its Discrimination from Closely Related Taxa.” *Mycologia* 104(5): 1121–32. http://www.mycologia.org/cgi/doi/10.3852/11-344.

Unlu, Ahmet, Erdinc Nayir, Onder Kirca, and Mustafa Ozdogan. 2016. “Ganoderma Lucidum (Reishi Mushroom) and Cancer.” *Journal of B.U.ON. : official journal of the Balkan Union of Oncology* 21(4): 792–98. http://www.ncbi.nlm.nih.gov/pubmed/27685898.

Vadivelan, Ganesan, and Govindarajulu Venkateswaran. 2014. “Production and Enhancement of Omega-3 Fatty Acid from Mortierella Alpina CFR-GV15: Its Food and Therapeutic Application.” *BioMed Research International* 2014: 1–9. https://www.hindawi.com/journals/bmri/2014/657414/.

Valente, Silvia et al. 2021. “CRISPR-Cas9-Based Discovery of the Verrucosidin Biosynthesis Gene Cluster in Penicillium Polonicum.” *Frontiers in Microbiology* 12(May): 1–11. https://www.frontiersin.org/articles/10.3389/fmicb.2021.660871/full.

Vizzini, Alfredo, Matteo Gelardi, Contu Marco, and Ming Zhang. 2014. “A New Collection of Chlorophyllum Hortense ( Agaricaceae , Agaricales ) from South-Eastern China : Molecular Confirmation and Morphological Notes.” (January).

Voto, P., F. Dovana, and M. Garbelotto. 2019. “A Revision of the Genus Psathyrella, with a Focus on Subsection Spadiceogriseae.” *Fungal Systematics and Evolution* 5(June): 119–29. https://www.ingentaconnect.com/content/10.3114/fuse.2019.04.08.

Wakefield, William S. et al. 2010. “A Molecular Phylogenetic Evaluation of the Spizellomycetales.” *Mycologia* 102(3): 596–604. https://www.tandfonline.com/doi/full/10.3852/09-120.

Wang, Meng Meng, Belle Damodara Shenoy, Wei Li, and Lei Cai. 2017. “Molecular Phylogeny of Neodevriesia, with Two New Species and Several New Combinations.” *Mycologia* 109(6): 965–74. https://doi.org/10.1080/00275514.2017.1415075.

Wang, Q.-M. et al. 2015. “Phylogenetic Classification of Yeasts and Related Taxa within Pucciniomycotina.” *Studies in Mycology* 81: 149–89. https://www.sciencedirect.com/science/article/pii/S0166061615000287.

Wang, Q. M. et al. 2015. “Phylogenetic Classification of Yeasts and Related Taxa within Pucciniomycotina.” *Studies in Mycology* 81: 149–89. http://dx.doi.org/10.1016/j.simyco.2015.12.002.

WANG, Q, and F BAI. 2004. “Four New Yeast Species of the Genus from Plant Leaves.” *FEMS Yeast Research* 4(6): 579–86. https://academic.oup.com/femsyr/article-lookup/doi/10.1016/j.femsyr.2003.11.002.

Wu, Jiang, Zhi-Ming Liao, and Peng-Cheng Lin. 2020. “CHEMICAL CONSTITUENTS OF Agaricus Gennadii.” *Chemistry of Natural Compounds* 56: 761+.

Wu, Sheng-Hua. 2001. “Three New Species of Hyphodontia with Poroid Hymenial Surface.” *Mycologia* 93(5): 1019–25. https://www.tandfonline.com/doi/full/10.1080/00275514.2001.12063235.

Xing, Xiaoying, Yuanhui Wang, Nairui Huo, and Rufu Wang. 2018. “*Candida Ethanolica* Strain Y18 Enhances Aroma of Shanxi Aged-Vinegar.” *Food Science and Technology Research* 24(6): 1069–81. https://www.jstage.jst.go.jp/article/fstr/24/6/24_1069/_article.

Yang, Hui-Xiang et al. 2018. “Trichothecrotocins A–C, Antiphytopathogenic Agents from Potato Endophytic Fungus Trichothecium Crotocinigenum.” *Organic Letters* 20(24): 8069–72. https://pubs.acs.org/doi/10.1021/acs.orglett.8b03735.

Yao, Yi-Jian et al. 2020. “On the Typification of Ganoderma Sichuanense (Agaricomycetes)− the Widely Cultivated Lingzhi Medicinal Mushroom.” *International Journal of Medicinal Mushrooms* 22(1).

Yilmaz, N. et al. 2014. “Polyphasic Taxonomy of the Genus Talaromyces.” *Studies in Mycology* 78(1): 175–341. http://dx.doi.org/10.1016/j.simyco.2014.08.001.

Yu, Changjun, Yucheng Dai, and Zhengquan Wang. 2004. “[A preliminary study on wood-inhabiting fungi on charred wood in Daxinganling forest areas].” *Ying yong sheng tai xue bao = The journal of applied ecology* 15(10): 1781–84. http://www.ncbi.nlm.nih.gov/pubmed/15624808.

Yurchenko, Eugene, Sheng-Hua Wu, and Nitaro Maekawa. 2020. “Three New Species of Peniophorella (Basidiomycota) from East Asia.” *Nova Hedwigia* 111(3–4): 473–95. http://dx.doi.org/10.1127/nova%5C_hedwigia/2020/0598.

Zhao, J.-H. 2003. “Sporobolomyces Bannaensis, a Novel Ballistoconidium-Forming Yeast Species in the Sporidiobolus Lineage.” *INTERNATIONAL JOURNAL OF SYSTEMATIC AND EVOLUTIONARY MICROBIOLOGY* 53(6): 2091–93. https://www.microbiologyresearch.org/content/journal/ijsem/10.1099/ijs.0.02807-0.
