# Supplementary material for: Size-resolved fungal bioaerosol diversity over an Indian agricultural field and their ecosystem-health implications
Source: Front Microbiol. 2025 Dec 3;16:1648820. doi: 10.3389/fmicb.2025.1648820 (PMC12708607; doi:10.3389/fmicb.2025.1648820)
Supplement: Supplementary file 3 [file Image_1.pdf]

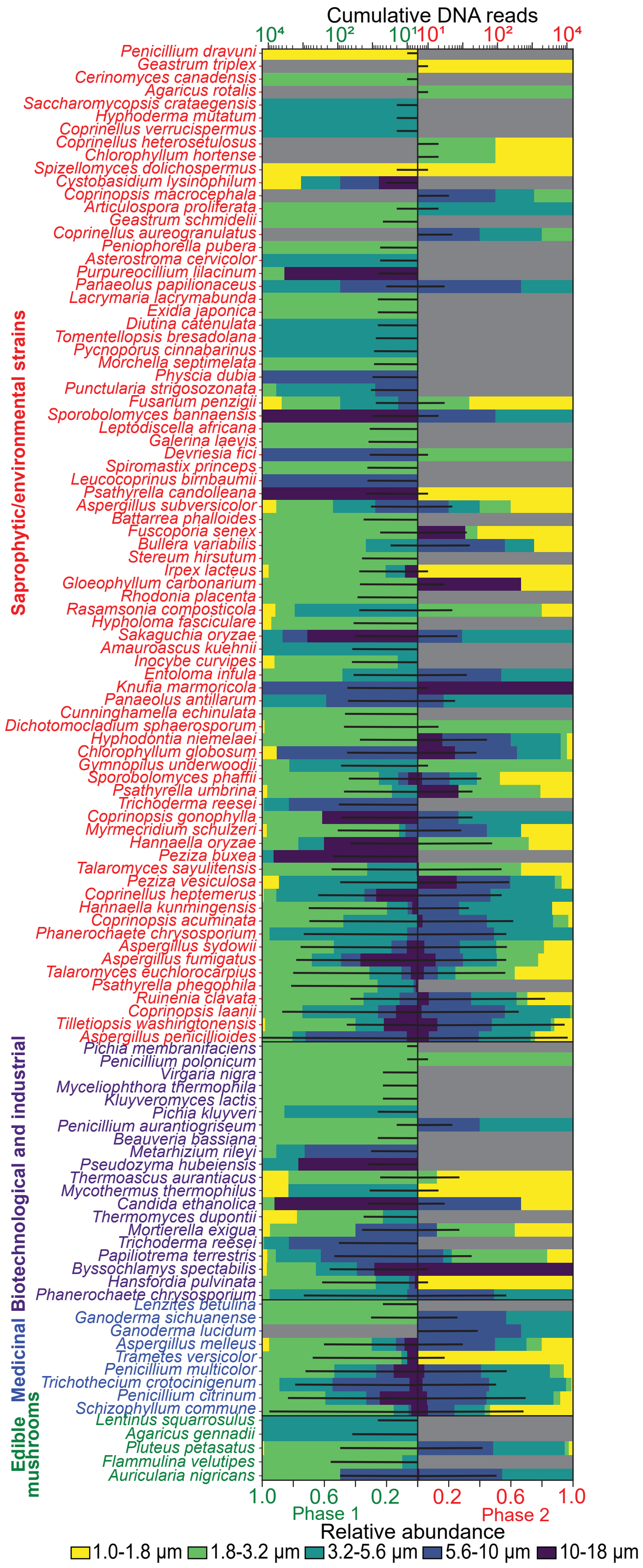

Supplementary Figure S1: Cummulative DNA sequnces and size-resolved relative abundance of the various beneficial/useful fungal species identified during the study.
